# Enteric glial cells favor accumulation of anti-inflammatory macrophages during the resolution of muscularis inflammation

**DOI:** 10.1038/s41385-022-00563-2

**Published:** 2022-09-07

**Authors:** Michelle Stakenborg, Saeed Abdurahiman, Veronica De Simone, Gera Goverse, Nathalie Stakenborg, Lies van Baarle, Qin Wu, Dimitri Pirottin, Jung-Seok Kim, Louise Chappell-Maor, Isabel Pintelon, Sofie Thys, Emilie Pollenus, Louis Boon, Philippe Van den Steen, Marlene Hao, Jo A. Van Ginderachter, Guy E. Boeckxstaens, Jean-Pierre Timmermans, Steffen Jung, Thomas Marichal, Sales Ibiza, Gianluca Matteoli

**Affiliations:** 1 Department of Chronic Diseases and Metabolism (CHROMETA), Translational Research Center for Gastrointestinal Disorders (TARGID), KU Leuven, Leuven, Belgium; 2 Laboratory of Cellular and Molecular Immunology, GIGA Institute, Liege University, Liege, Belgium; 3 Department of Immunology, Weizmann Institute of Science, Rehovot, Israel; 4 Laboratory of Cell Biology & Histology, Department of Veterinary Sciences, University of Antwerp, Antwerp, Belgium; 5 Laboratory of Immunoparasitology, Department of Microbiology, Immunology and Transplantation, Rega Institute for Medical research, KU Leuven, Leuven, Belgium; 6 Polpharma Biologics, Utrecht, the Netherlands; 7 Cellular and Molecular Immunology Lab, Department of Bio-engineering Sciences, Vrije Universiteit Brussel, Brussels, Belgium; 8 Myeloid Cell Immunology Lab, VIB Center for Inflammation Research, Brussels, Belgium; 9 Laboratory of Immunophysiology, GIGA Institute, Liege University, Liege, Belgium; 10 Department of Functional Sciences, Faculty of Veterinary Medicine, Liege University, Liege, Belgium

## Abstract

Monocyte-derived macrophages (Mφs) are crucial regulators during muscularis inflammation. However, it is unclear which micro-environmental factors are responsible for monocyte recruitment and anti-inflammatory Mφ differentiation in this paradigm. Here, we investigate Mφ heterogeneity at different stages of muscularis inflammation and determine how environmental cues can attract and activate tissue-protective Mφs. Results showed that muscularis inflammation induced marked alterations in mononuclear phagocyte populations associated with a rapid infiltration of Ly6c^+^ monocytes that locally acquired unique transcriptional states. Trajectory inference analysis revealed two main pro-resolving Mφ subpopulations during the resolution of muscularis inflammation, i.e. Cd206^+^ MhcII^hi^ and Timp2^+^ MhcII^lo^ Mφs. Interestingly, we found that damage to the micro-environment upon muscularis inflammation resulted in EGC activation, which in turn stimulated monocyte infiltration and the consequent differentiation in anti-inflammatory CD206^+^ Mφs via CCL2 and CSF1, respectively. In addition, CSF1-CSF1R signaling was shown to be essential for the differentiation of monocytes into CD206^+^ Mφs and EGC proliferation during muscularis inflammation. Our study provides a comprehensive insight into pro-resolving Mφ differentiation and their regulators during muscularis inflammation. We deepened our understanding in the interaction between EGCs and Mφs, thereby highlighting pro-resolving Mφ differentiation as a potential novel therapeutic strategy for the treatment of intestinal inflammation.

## Introduction

In recent years, significant advances have been made in our understanding of the phenotype of intestinal macrophage (Mφ) archeotypes, which perform a variety of niche-defined functions from preserving immune homeostasis to regulating motility and secretion via crosstalk with the enteric nervous system (ENS)^1–3^. Consequently, specific intestinal niches are populated by different Mφ subsets with a unique transcriptome to meet the diverse functional demands of the tissue micro-environment. For instance, lamina propria Mφs reside in close proximity to the epithelium and at the crypt base, where they are involved in the maintenance of gut homeostasis by contributing to epithelial barrier integrity and by phagocytosing luminal antigens^2–5^. In the deeper layers of the gut wall at the level of the muscularis externa, Mφs exert neurotrophic functions promoting enteric neuronal survival and supporting neuronal function^1,6,7^. However, during muscularis inflammation, activation of resident Mφs results in impairment of neuromuscular contractility due to the release of nitric oxide, prostaglandins and cytokines, simultaneously triggering the influx of inflammatory cells^8–10^. Although we have shown in a previous study that the influx of monocytes is essential for the resolution of muscularis inflammation due to their differentiation into pro-resolving monocyte-derived Mφs^11^, it remains to be determined which cell types are responsible for monocyte recruitment and which factors promote their differentiation towards anti-inflammatory Mφs during muscularis inflammation. The extensive crosstalk between muscularis Mφs and the ENS at homeostasis suggests a potential involvement also during inflammation^6^. The ENS is organized in a network of ganglia containing neurons and enteric glial cells (EGCs). Historically, EGCs were mainly considered as supporting cells of enteric neurons. However, recent evidence suggests that EGCs have a much broader function in gastro-intestinal (GI) physiology, contributing to motility and preserving epithelial barrier integrity by the interaction with innate lymphoid cells (ILCs), interstitial cells of Cajal, endothelial and epithelial cells^12–17^. Even more intriguing is the putative role of EGCs in immune regulation. In this regard, the interaction between astrocytes and microglia has been extensively studied in the brain during development, homeostasis and disease^18^. Yet, limited studies have investigated the interaction between Mφs and EGCs in the intestine^19^.

Here, we investigate Mφ heterogeneity and their role in tissue repair during muscularis inflammation. Using time-series single-cell transcriptomics, we observed that muscularis inflammation induced a prominent myeloid cell diversification, resulting in 7 myeloid subpopulations. Trajectory inference analysis indicated that incoming Ly6c^hi^ monocytes acquired diverse gene expression signatures in the injured muscularis, notably resulting in two main pro-resolving Mφ subpopulations characterized by Cd206^+^ MhcII^hi^ and Timp2^+^ MhcII^lo^ gene signatures. In addition, EGCs were identified as the main producers of the monocyte chemoattractant, CCL2, during the early phase of inflammation. Furthermore, these cells produced a variety of secreted factors that can potentially stimulate the differentiation of monocytes into pro-resolving Mφs. Specifically, we found that EGC-derived CSF1 is a critical signal for the generation of anti-inflammatory CD206^+^ Mφs in vitro and that blocking CSF1R in vivo diminishes CD206^+^ Mφ differentiation leading to damage to the EGCs. Overall, our findings imply that during intestinal inflammation, future therapies should be aimed at enhancing the pro-resolving and neurotrophic properties of Mφs to reduce possible damage to the ENS and promote functional GI recovery.

## Results

### Time-dependent recruitment of myeloid cells in the inflamed muscularis

**Fig. 1:**
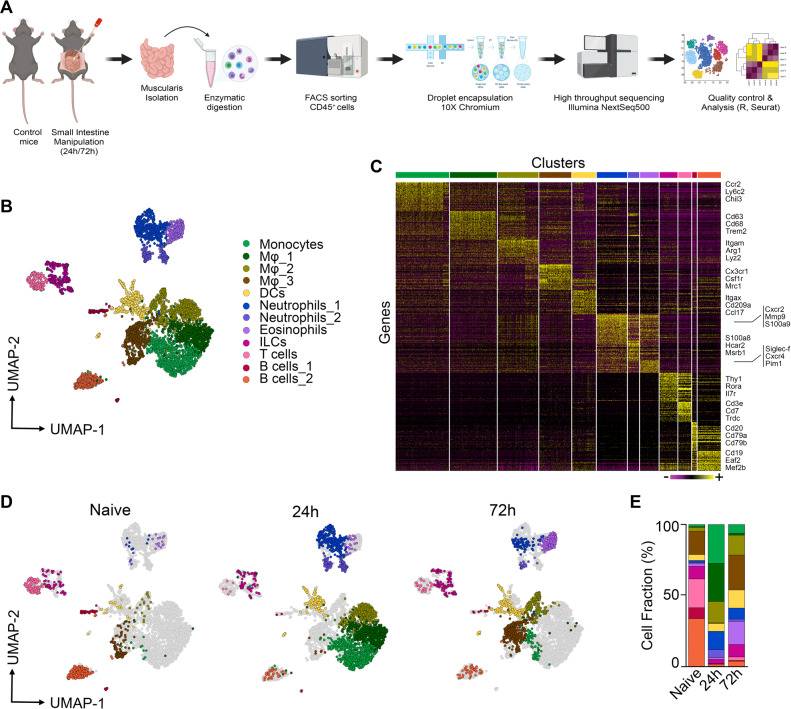
Identification of CD45^+^ immune cell populations by unsupervised scRNA-seq clustering in the healthy and inflamed muscularis. **a** Experimental pipeline of scRNA-seq experiment. **b** UMAP of sorted CD45^+^ immune cells from the healthy muscularis, 24 h and 72 h post-injury from WT mice. Each sample was pooled from 3–4 mice. **c** Heatmap of the 50 most differentially expressed genes in each cluster. **d** UMAPs of time-dependent infiltration of immune cells upon muscularis inflammation. Data of Fig. 1**b** has been split based on different time points. **e** Cell fraction of each cluster relative to the total number of CD45^+^ immune cells at different time points after muscularis inflammation.

Surgery-induced damage to the muscularis leads to a transient impairment of GI motility associated with extensive recruitment of immune cells to the ENS. To characterize the immune cell infiltrate and their respective activation states during muscularis inflammation, droplet-based single cell RNA sequencing (scRNA-seq) was performed on sorted CD45^+^ immune cells from the muscularis of naïve mice, and during the acute inflammatory (24 h) and the recovery phase of muscularis inflammation (72 h) (10X Genomics Platform; Fig. 1a). Unsupervised clustering of 4102 cells and reference-based cell identification using Immgen (Fig. 1b, c and Supplementary Fig. 1a, b) revealed 12 independent immune cell populations including monocytes (*Ccr2, Ly6c2, Chil3*), 3 clusters of Mφs (1: *Cd63, Cd68, Trem2*; 2: *Itgam, Arg1, Lyz2*; 3: *Cx3cr1, Csf1r, Mrc1*), dendritic cells (DCs; *Itgax, Cd209a, Ccl17*), 2 clusters of neutrophils (1: *Cxcr2, Mmp9, S100a9*; 2:, *S100a8, Hcar2, Msrb1*), eosinophils (*Siglec-f, Cxcr4, Pim1*), ILCs (*Thy1, Rora, Il7r*), T cells (*Cd3e, Cd7, Trdc*) and 2 clusters of B cells (1: *Cd79a, Cd20, CD79b*; 2: *Eaf2, Mef2b, CD19*)^20^.

To examine the role of different immune cell populations, we determined their gene expression signatures at different time points during muscularis inflammation. At homeostasis, the muscularis mainly consisted of a population of Cx3cr1^+^ Mφs (Mφ_3) together with T cells, B cells and ILCs (Fig. 1d, e). Consistent with previous observations^11,21^, a shift in the leukocyte populations was observed in the acute phase (24 h) after the induction of muscularis inflammation with a massive infiltration of monocytes in addition to Mφs (Mφ_1 and Mφ_2) and neutrophils (Neu_1 and Neu_2), pointing towards a pro-inflammatory micro-environment (Fig. 1d, e). During the resolution of muscularis inflammation (72 h), the muscularis was mainly populated by Cx3cr1^+^ Mφs (Mφ_3), DCs and eosinophils, indicating a return to homeostasis. Altogether, these results show that during muscularis inflammation a shift in the immune landscape favors the resolution of inflammation and a rapid return to homeostasis.

### Identification of two distinct myeloid subpopulations during the resolution of muscularis inflammation

**Fig. 2:**
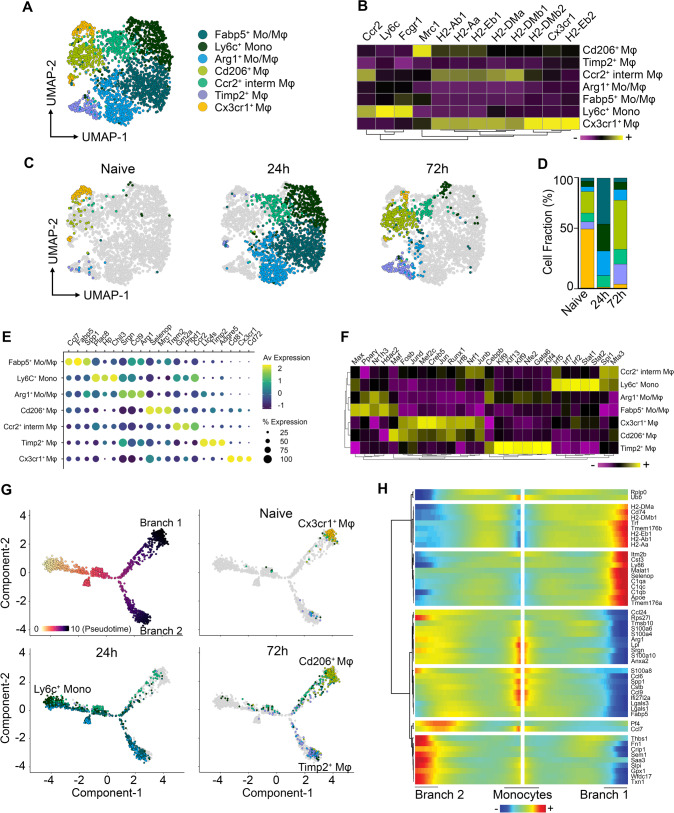
Two main anti-inflammatory Mφ subpopulations with a unique transcriptional state are present during the resolution of muscularis inflammation. **a** UMAP of reclustered monocyte/Mφ subpopulations from Fig. 1a from the healthy muscularis, 24 h and 72 h post-injury from WT mice. **b** Heatmap of typical monocyte and Mφ markers including MHCII genes. **c** UMAPs of time-dependent infiltration of monocyte and Mφ subsets upon muscularis inflammation. **d** Cell fraction of each subcluster relative to the total number of monocytes/Mφs at different time points after muscularis inflammation. **e** Dotplot showing expression of selected differentially expressed genes in each subcluster. **f** Heatmap of regulon activity per cluster according to SCENIC analysis. **g** Pseudotime analysis of monocytes/Mφs at different time points after muscularis inflammation. **h** Heatmap of gene expression showing the top 50 genes of different branches of the pseudotime trajectory tree.

Monocyte-derived Mφs are essential for the recovery of GI motility during the resolution of muscularis inflammation^11,21^. However, their heterogeneity and differentiation trajectory towards tissue-protective Mφs have not yet been characterized during muscularis inflammation. To this end, subsets originally identified as monocyte/Mφ subpopulations in our scRNA-seq dataset (Fig. 1) were extracted and re-clustered to better define which types of myeloid cells might aid in the resolution of muscularis inflammation, thereby identifying 7 distinct subsets (Fig. 2a–d and Supplementary Fig. 2a). At homeostasis, the muscularis micro-environment mainly consisted of Cx3cr1^+^ Mφs with high expression of typical resident Mφ markers such as *Cd81*, *Cd72* and *H2-Eb1* (Fig. 2e). During acute muscularis inflammation, the most predominant subpopulation was the cluster of classical Ly6c^+^ monocytes, which was enriched for the expression of *Plac8*, *Hp* and *Chil3* (Fig. 2b–e). Additionally, a subpopulation with a gene expression signature suggestive of an intermediate monocyte-to-Mφ differentiation state was observed (Ccr2^+^ int Mφs), which was underscored by their moderate *MhcII* expression as compared to Ly6c^+^ monocytes and homeostatic Cx3cr1^+^ Mφs (Fig. 2b). Besides Ly6c^+^ monocytes and Ccr2^+^ int Mφs, we observed a subcluster of Arg1^+^ Mo/Mφs with high expression of *Ccl9* and *Srgn* alongside Fabp5^+^ Mo/Mφs with characteristic expression of *Lgals1*, *Ccl7* and *Flt1* (Fig. 2e). Of note, 72 h post-injury, during the resolution of muscularis inflammation, Ly6c^+^ monocytes and Ccr2^+^ int Mφs were present at a low percentage but two novel Mφ subclusters were identified: Cd206^+^ Mφs and Timp2^+^ Mφs (Fig. 2c, d and Supplementary Fig. 2b). The most abundant subcluster during the resolution of muscularis inflammation, Cd206^+^ Mφs, displayed high *MhcII* expression similar to homeostatic Cx3cr1^+^ Mφs, in addition to high expression of anti-inflammatory genes such as *Selenop*, *Mrc1*, *Igf1*, *Trem2* and *Stab1* (Fig. 2b and Supplementary Fig. 2b, c). In contrast, Timp2^+^ Mφs had lower *MhcII* expression compared to homeostatic Cx3cr1^+^ Mφs, but similarly expressed high levels of tissue reparative markers such as *Ltc4s* and *Adgre5* (Fig. 2b, e and Supplementary Fig. 2b, c). To further investigate differences in the transcriptional regulation of Cd206^+^ and Timp2^+^ Mφs, single-cell regulatory network inference and clustering (SCENIC) was employed to assess specific regulon activities in different myeloid subclusters (Fig. 2f and Supplementary Fig. 2f). Cd206^+^ Mφs displayed high regulon activity of *FosB, Runx1* and *Irf8* similar to Cx3cr1^+^ Mφs, while Timp2^+^ Mφs had a distinctly altered transcription factor signature with high *Cebpb* and *Gata6* regulon activity.

To evaluate whether these subclusters can also be detected by flow cytometry, a comparative study was performed during muscularis inflammation. In the healthy muscularis, only 1 subset of MHCII^hi^ Mφs was observed similar to homeostatic Cx3cr1^+^ Mφs (Supplementary Fig. 2d, e). During acute muscularis inflammation, the muscularis micro-environment was mainly dominated by infiltrating Ly6C^hi^ monocytes, which differentiated into Ly6C^+^ MHCII^+^ immature Mφs. However, at single cell level, we observed 4 different Mφ subclusters, indicating that Mφ heterogeneity was higher than originally considered. Interestingly, 72 h post-injury, flow cytometric analysis also revealed only two main Mφ subclusters (MHCII^hi^ and MHCII^lo^ Mφs), similar to our observations at single cell level (Supplementary Fig. 2d, e).

Identifying relevant gene expression changes in myeloid subclusters during muscularis inflammation could shed light on the molecular mechanisms regulating Mφ differentiation. In order to define these possible temporal transcriptional alterations leading to differentiation into Cd206^+^ and Timp2^+^ Mφs, Monocle-2 was used to superimpose subclusters on a trajectory placing Ly6c^+^ monocytes at the beginning of the pseudotime (Fig. 2g, h)^22^. Using this approach, we identified a major trajectory bifurcation leading to a branch of cells with high expression of genes found in homeostatic Mφs such as *Cd74*, *Tmem176a/b*, complement genes (*C1qa, C1qb, C1qc*) and *MhcII* genes (*H2-Eb1, H2-Ab1, H2-Aa*; Branch 1), while the cells in the second branch expressed less complement and *MhcII* genes but had increased expression of *Fn1, Ltc4s* and *Saa3* (Branch 2) (Fig. 2h and Supplementary Fig. 2g, h). Interestingly, the Cd206^+^ and Timp2^+^ Mφ subsets were at two opposite ends of the trajectory suggesting different differentiation paths during the resolution of muscularis inflammation (Fig. 2g). Of note, homeostatic Cx3cr1^+^ Mφs were located in close proximity to Cd206^+^ Mφs in the pseudotime, underscoring the similarity between both Mφ subsets. Arg1^+^ and Fabp5^+^ Mo/Mφs were mainly present in Branch 2, while Ccr2^+^ int Mφs were exclusively present in Branch 1, indicating that these subpopulations might act as intermediates before attaining their terminal differentiation state. These findings support the hypothesis that there are two major monocyte-to-Mφ differentiation trajectories in the inflamed muscularis giving rise to Cd206^+^ and Timp2^+^ Mφs during the resolution of inflammation.

### Pro-resolving Mφs originate from CCR2^±^ monocytes during muscularis inflammation

**Fig. 3:**
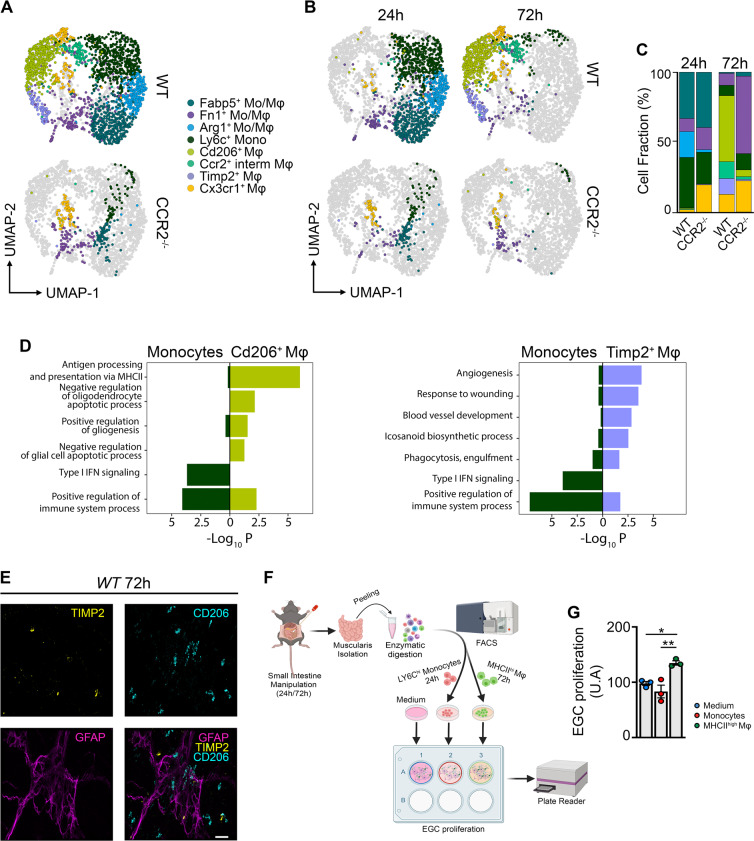
Two distinct Mφ subpopulations during the resolution of muscularis inflammation are derived from CCR2^+^ monocytes. **a** UMAP of monocyte and Mφ subclusters from the muscularis of WT and CCR2^−/−^ mice at 24 h and 72 h after induction of muscularis inflammation. Each sample was pooled from 3–4 mice. **b** UMAPs of myeloid cells at different time points post-injury. **c** Cell fraction of each subcluster relative to the total number of myeloid cells at different time points after muscularis inflammation. **d** GO analysis of monocytes versus Cd206^+^ Mφs (left) or Timp2^+^ Mφs (right) showing negative Log_10_(*p*-value). **e** Immunofluorescent images of muscularis whole-mount preparations 3 days after the induction of muscularis inflammation stained for GFAP (purple), TIMP2 (yellow) and CD206 (light blue). Scale bar 15 µm. **f** Experimental outline of in vitro EGC proliferation by stimulation with supernatant of monocytes or Mφs from different time points post-injury. **g** Fold induction of EGCs stimulated with the supernatant of LY6C^hi^ monocytes from 24 h post-injury or MHCII^hi^ Mφs from 72 h post-injury relative to control medium. Every data point is an independent sorting and culture experiment One-way ANOVA; test *p < 0.05; **p < 0.01; ns not significant.

In a previous study, we have shown that the influx of CCR2^+^ monocytes is essential for resolution of muscularis inflammation, as blocking monocyte infiltration resulted in a delayed recovery of GI motility and damage to the ENS^11^. Thus, to investigate whether CCR2^+^ monocytes are the source of pro-resolving Mφs during muscularis inflammation, scRNA-seq was performed on sorted CD45^+^ immune cells from the muscularis of WT and CCR2^−/−^ mice 24 h and 72 h post-injury (Fig. 3a–c and Supplementary Fig. 3a–d). After extraction and re-clustering of the monocyte and Mφ subsets (2,406 cells), the gene expression signatures from the myeloid subsets in Fig. 2 were crossmatched by singleR with this novel dataset to specifically annotate the subpopulations (Supplementary Fig. 3c), yielding only one additional cluster, i.e., Fn1^+^ Mo/Mφs. In CCR2^−/−^ mice, there was a large alteration in the myeloid compartment after muscularis inflammation. As expected upon acute muscularis inflammation in CCR2^−/−^ mice, there was an almost complete loss of Ly6c^+^ monocytes, and Arg1^+^ and Fabp5^+^ Mo/Mφs. In addition, we could only detect a small subpopulation of Cx3cr1^*+*^ Mφs representing the long-lived resident Mφs. During the resolution of muscularis inflammation, the Cd206^+^ and Timp2^+^ Mφ subsets were also completely absent in CCR2^−/−^ mice, confirming that these subpopulations are derived from CCR2^+^ monocytes and that they possibly represent the pro-resolving Mφs essential for recovery of GI motility after tissue damage.

To further investigate the phenotype of Cd206^+^ and Timp2^+^ Mφs during muscularis inflammation, gene ontology (GO) analysis was performed and revealed that both Mφ subsets seemed pro-resolving in nature (Fig. 3d). For instance, Timp2^+^ Mφs were implicated in pathways associated with tissue repair and regulation of the inflammatory response, such as angiogenesis, response to wounding, blood vessel development, icosanoid biosynthetic processes, and phagocytosis-engulfment. Conversely, Cd206^+^ Mφs were enriched for pathways associated with antigen processing and presentation via MHC class II, negative regulation of oligodendrocyte and glial cell apoptotic processes and positive regulation of gliogenesis. In contrast, monocytes were clearly enriched for pro-inflammatory pathways, such as type I interferon signaling and positive regulation of immune system processes. Interestingly, CD206^+^ Mφs were located in close proximity or even surrounded the ENS and outnumbered TIMP2^+^ Mφs, which were located further away from the ENS (Fig. 3e). To confirm that these Cd206^+^ MhcII^hi^ Mφs have a neuroprotective effect on the ENS, we nassessed their contribution to EGC proliferation in vitro (Fig. 3f). While Ly6c^+^ monocytes isolated 24 h post-injury did not alter the proliferation of EGCs, MHCII^hi^ Mφs from 72 h post-injury significantly induced EGC proliferation, underscoring their role in supporting EGC function (Fig. 3g). These results establish that CCR2^+^ monocytes differentiate into two distinct Mφ subpopulations with pro-resolving and/or neurotrophic functions.

### CX3CR1^GFP^-based mapping matches unique Mφ subsets observed in scRNA-seq during muscularis inflammation

**Fig. 4:**
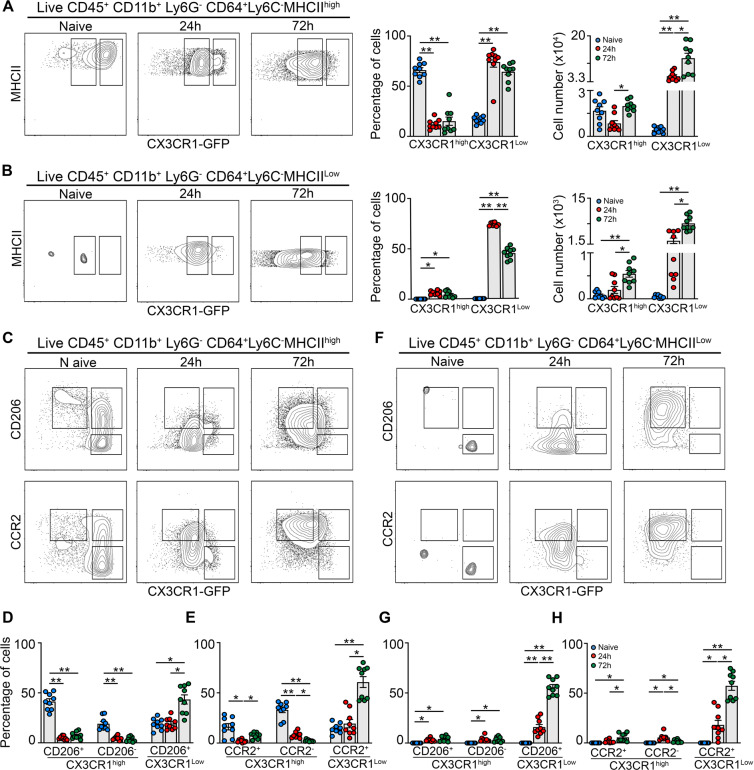
Flow cytometry validates unique Mφ subpopulations during muscularis inflammation. **a–h** from naïve CX3CR1^GFP/+^ mice, 24 h and 72 h after muscularis inflammation. **a** Contour plots representing CX3CR1 expression in Ly6C^−^ MHCII^hi^ Mφs (left). Percentages and absolute numbers of CX3CR1^hi^ and CX3CR1^lo^ cells from Ly6C^−^ MHCII^hi^ Mφs are shown as mean ± SEM (right). **b** Contour plots representing CX3CR1 expression in Ly6C^−^ MHCII^lo^ Mφs (left). Percentages and absolute numbers of CX3CR1^hi^ and CX3CR1^lo^ cells from Ly6C^−^ MHCII^lo^ Mφs are shown as mean ± SEM (right). **c**–**h** CD206 and CCR2 expression in Ly6C^−^ MHCII^hi^ and MHCII^lo^ Mφs. Contour plots representing CD206 or CCR2 expression in Ly6C^−^ MHCII^hi^ (**c**) and Ly6C^−^ MHCII^lo^ Mφs (**f**). Percentages of CD206^+^ CX3CR1^hi^, CD206^−^ CX3CR1^hi^ and CD206^+^ CX3CR1^lo^ cells in the LY6C^−^ MHCII^hi^ Mφs (**d**) and MHCII^lo^ Mφs (**g**). Percentages of CCR2^+^ CX3CR1^hi^, CCR2^−^ CX3CR1^hi^ and CCR2^+^ CX3CR1^lo^ cells in the LY6C^−^ MHCII^hi^ Mφs (**e**) and MHCII^lo^ Mφs (**h**). One-way ANOVA; test *p < 0.05; **p < 0.01; ns not significant.

To further define the phenotype of the Mφ subpopulations identified using scRNA-seq at the protein level, we induced muscularis inflammation in CX3CR1^gfp/+^ mice (Fig. 4a, b). Upon muscularis inflammation, the percentage of homeostatic CX3CR1^hi^ Mφs was drastically reduced with a concomitant increase of CX3CR1^lo^ MHCII^hi^ and CX3CR1^lo^ MHCII^lo^ Mφs. As CCR2 and CD206 have been described as essential markers for recruited and anti-inflammatory Mφs respectively, their protein levels were determined in these CX3CR1^hi/lo^ Mφ subpopulations (Fig. 4c–h and Supplementary Fig. 4a, b). In line with previous findings, CX3CR1^hi^ MHCII^hi^ Mφs were mainly CD206^+^ and CCR2^−^ at homeostasis (Fig. 4c–e). However, this subpopulation was gradually replaced by a combination of CX3CR1^lo^ MHCII^lo^ and CX3CR1^lo^ MHCII^hi^ Mφs, resembling Timp2^+^ and Cd206^+^ Mφs from our scRNA-seq dataset, respectively (Fig. 4c–h). Interestingly, CD206^+^ CX3CR1^lo^ MHCII^hi^ Mφs 10-fold outnumbered CX3CR1^lo^ MHCII^lo^ Mφs, further underscoring the importance of these anti-inflammatory CD206^+^ MHCII^hi^ Mφs (Supplementary Fig. 4a, b). Overall, our data validated at protein level the presence of two distinct Mφ subpopulations during the resolution of muscularis inflammation which are derived from CCR2^+^ monocytes and are anti-inflammatory in nature.

In line with our scRNA-seq datasets, we observed a drastic reduction in the percentage of homeostatic CX3CR1^hi^ Mφs upon the induction of muscularis inflammation (Fig. 2c, d). To determine whether these CX3CR1^hi^ Mφs are replaced by incoming monocyte-derived Mφs, CX3CR1^+^ resident Mφs were mapped by using a tamoxifen-inducible CX3CR1^CreERT2^ strain backcrossed with Rosa26-LSL-YFP mice. By comparing circulating unlabeled blood monocytes with resident YFP^+^ Mφs, we could show that resident Mφs did not diminish in number during muscularis inflammation as quantified by immunofluorescence and flow cytometry (Supplementary Fig. 4c–f). Taken together, these results indicate that CX3CR1^hi^ Mφs were present alongside CX3CR1^lo^ mature Mφs during muscularis inflammation.

### Damage-activated EGCs initiate the recruitment and differentiation of monocytes upon muscularis inflammation

**Fig. 5:**
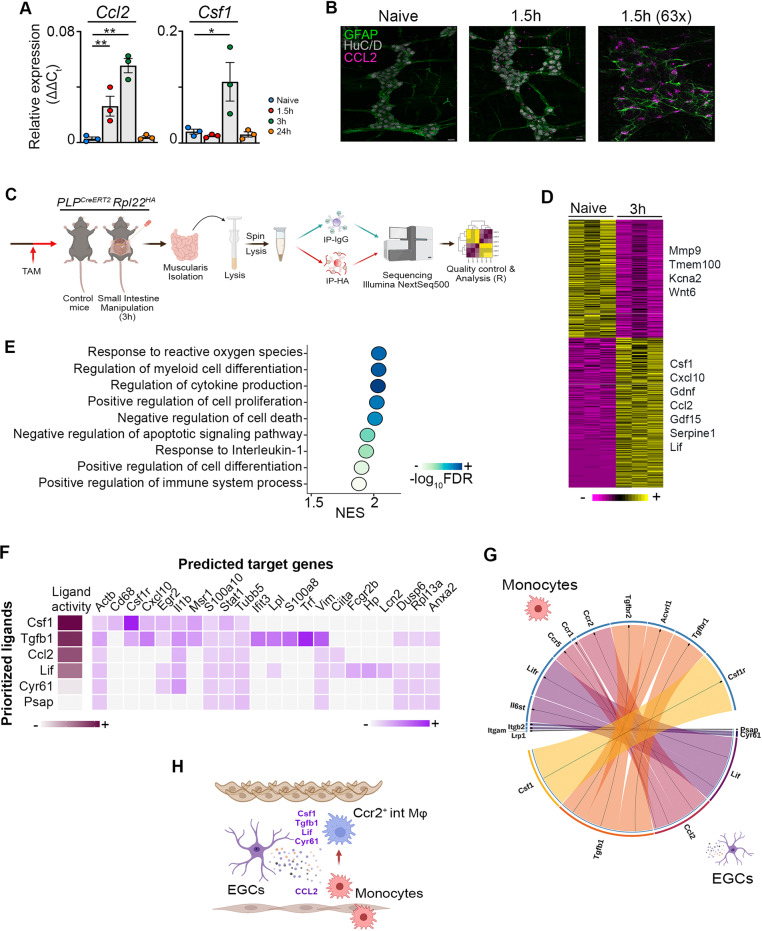
EGCs produce factors essential for the recruitment and differentiation of monocytes during inflammation. **a** Relative mRNA levels for Ccl2 and Csf1 normalized to the housekeeping gene *rpl32* from ganglia isolated from the muscularis of the small intestine from naïve wild-type mice and 1.5 h, 3 h and 24 h after muscularis inflammation. One-way ANOVA; test *p < 0.05; **p < 0.01; ns not significant. **b** Immunofluorescent images of muscularis whole-mount preparations at homeostasis and 1.5 h after the induction of muscularis inflammation stained for GFAP (green), HuC/D (gray) and CCL2 (purple). Scale bar (25x) 25 µm, (63x) 15 µm. **c** Experimental overview of experiments in PLP-CreERT2 Rpl22^HA^ mice. **d** Heatmap of HA-enriched differentially expressed genes between immunoprecipitated samples from naïve PLP-CreERT2 Rpl22^HA^ mice and 3 h after intestinal manipulation. **e** Selected significant GO terms enriched (GSEA) in PLP1^+^ EGCs 3 h post-injury compared to naive PLP1^+^ EGCs. **f** Heat map of ligand-target pairs showing regulatory potential scores between top positively correlated prioritized ligands and their target genes among the differentially expressed genes between Ly6c^+^ monocytes and Ccr2^+^ int Mφs. **g** Circos plot showing top NicheNet ligand-receptor pairs between EGCs and Ly6c^+^ monocytes corresponding to the prioritized ligands in Fig. 5**f**. **h** Schematic overview of interactions between EGCs and infiltrating Ly6c^+^ monocytes.

Monocytes originate from progenitors in the bone marrow and rely on local chemokine production such as MCP-1/CCL2 for their recruitment at the site of inflammation^23^. Considering the extensive interaction between CCR2^+^ monocytes and the ENS in the inflamed muscularis^6,11^, we examined the production of the major chemoattractant Ccl2 in the muscularis during the early stage of surgery-induced inflammation. Analysis of isolated enteric ganglia 1.5 h post-injury showed an increased gene expression of *Ccl2* and *Csf1* (Fig. 5a). Moreover, immunofluorescent images showed that CCL2 was specifically produced by GFAP^+^ EGCs (Fig. 5b), leading us to conclude that during the early stages of muscularis inflammation, EGCs likely initiate the recruitment of monocytes via CCL2 to stimulate tissue repair.

To further explore the role of EGCs during the early phase of muscularis inflammation, PLP^CreERT2^ Rpl22^HA^ mice were used, which allow immunoprecipitation (IP) of ribosome‐bound mRNAs from PLP1^+^ EGCs to study the transcriptome of EGCs at homeostasis and during early inflammation (3 h post-injury) (Fig. 5c and Supplementary Fig. 5a, b)^24–26^. Unlike pro-inflammatory reactive astrocytes during brain injury^27^, EGCs were activated early after muscularis inflammation and produced increased amounts of factors that are able to promote the differentiation of anti-inflammatory Mφs, such as *Lif, Serpine-1, Gdf15, Gdnf, Cxcl10* and *Csf1* (Fig. 5d and Supplementary Fig. 5c). To explore which pathways are activated in EGCs, gene set enrichment analysis was performed. Positive normalized enrichment scores were identified for response to ROS and cytokines including IL-1, indicating that EGCs are able to sense tissue damage in the muscularis micro-environment to initiate an appropriate immune response (Fig. 5e). These results were further underscored by showing that neurosphere-derived EGCs stimulated with IL1-α and/or IL1-β produced increased amounts of *Ccl2* compared to unstimulated EGCs (Supplementary Fig. 5d). Interestingly, there was also an overrepresentation of genes involved in the regulation of myeloid differentiation during early muscularis inflammation in EGCs (Fig. 5e and Supplementary Fig. 5e). These results suggest that activation of EGCs by cytokines including IL-1 could lead to both the recruitment and differentiation of monocytes during the early stages of muscularis inflammation.

To determine whether EGCs are able to stimulate monocyte differentiation during muscularis inflammation, we analyzed the expression of ligand-receptor pairs based on differentially expressed genes in PLP1^+^ EGCs 3 h post-injury and Ly6c^+^ monocytes undergoing differentiation into Ccr2^+^ int Mφs. Their expression data was combined with prior knowledge of signaling and gene regulatory networks using Nichenet^28^. To identify genes regulated by the identified ligands, putative ligand-gene interactions were scored by NicheNet according to their “regulatory potential” (Fig. 5f). We next assessed whether the expression of the receptors for the putative ligand-receptor interaction were altered when Ly6c^+^ monocytes underwent differentiation into Ccr2^+^ int Mφs (Supplementary Fig. 5f–h). By determining a threshold for the receptor expression, we identified the following ligand-receptor interactions between EGCs and differentiating monocytes: *Csf1-Csf1r, Tgfb1-Tgfbr2/Acvrl1/Tgfbr1, Ccl2-Ccr5/Ccr1/Ccr2, Lif-Lifr/Il6st, Cyr61-Itgam/Itgb2, and Psap-Lrp1* (Fig. 5g and Supplementary Fig. 5g). Taken together, damage to the muscularis micro-environment leads to activation of EGCs, that are able to recruit monocytes to the site of inflammation via CCL2 and that produce factors that have the potential to promote anti-inflammatory Mφ differentiation (Fig. 5h).

### EGCs promote the differentiation of monocytes into pro-resolving CD206^±^ Mφs in vitro

**Fig. 6:**
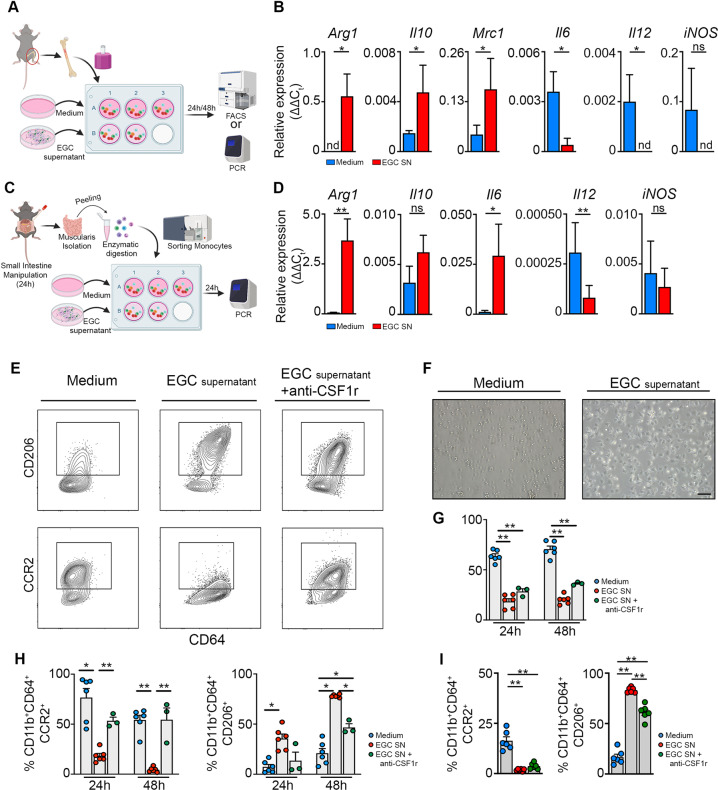
EGCs stimulate the differentiation of monocytes into anti-inflammatory CD206^+^ Mφs in part via CSF-1 in vitro. **a** Experimental outline of in vitro primary bone marrow monocytes stimulated with supernatant of EGCs. **b** Bone marrow-derived monocytes were stimulated for 24 h with/without supernatant of EGCs. Relative mRNA levels for pro- and anti-inflammatory cytokines normalized to the housekeeping gene *rpl32* in bone marrow monocytes cultured with/without EGC supernatant. **c** Experimental outline of in vitro experiment using sorted Ly6C^+^ MHCII^−^ monocytes stimulated with/without EGC supernatant for 24 h. **d** Ly6C^+^ MHCII^−^ monocytes were sorted from the muscularis of WT mice 24 h after the induction of muscularis inflammation and were stimulated for 24 h with/without supernatant of EGCs. Relative mRNA levels of pro- and anti-inflammatory mediators normalized to the housekeeping gene *rpl32* in sorted Ly6C^+^ MHCII^−^ monocytes stimulated with/without EGC supernatant. **e**–**h** Bone marrow monocytes were cultured for 24–48 h with/without EGC supernatant and supplemented with anti-CSF1r antibody. **e** Contour plots of bone marrow monocytes showing expression of CD206 (top) or CCR2 (bottom) upon culture for 48 h with/without EGC supernatant and supplemented with anti-CSF1r antibody. **f** Brightfield images of monocytes upon culture for 24 h with/without EGC supernatant. **g** Quantification of 7-AAD^+^ cells in bone marrow monocytes cultured for 24 h or 48 h with/without EGC supernatant and supplemented with anti-CSF1r antibody. **h** Percentages of CCR2^+^ and CD206^+^ cells in live CD45^+^ CD11b^+^ Ly6G^−^ CD64^+^ population. **i** Ly6C^+^ MHCII^−^ monocytes were sorted from the muscularis of WT mice 24 h after the induction of muscularis inflammation and were cultured with/without EGC supernatant and supplemented with anti-CSF1r antibody for 48 h. Percentages of CCR2^+^ and CD206^+^ cells in live CD45^+^ CD11b^+^ Ly6G^−^ CD64^+^ population. **a**–**d** *T*-test. **g**–**i** one-way ANOVA. *p < 0.05; **p < 0.01.

To directly assess the functional interaction between EGCs and monocytes, neurosphere-derived EGCs were analyzed by bulk RNAseq. The results revealed high expression of cytokines involved in the induction of anti-inflammatory Mφ differentiation (Supplementary Fig. 6a–c), similar to those observed in vivo in EGCs, including *Lif, Csf1, Tgfb1* and *Ccl2* (Fig. 5d, f). To demonstrate that EGC-derived factors are able to stimulate monocyte differentiation, an ex vivo model was set-up to co-culture bone marrow-derived monocytes with the supernatant of EGCs (Fig. 6a)^29^. Co-culture of bone marrow monocytes with EGC supernatant resulted in an upregulation of several anti-inflammatory markers such as *Arg1, Il10* and *Mrc1*, while pro-inflammatory markers were downregulated, including *Il6* and *Il12* (Fig. 6b). In addition, when bone marrow monocytes were exposed to bacterial components (LPS), a similar anti-inflammatory effect of EGC supernatant on monocytes (EGCs+LPS) was observed compared to LPS alone on monocytes (Supplementary Fig. 6d). Finally, to confirm that muscularis monocytes responded to EGC supernatant in a similar manner as bone marrow monocytes given their different origin, ex vivo experiments were performed on sorted Ly6C^+^ monocytes from the muscularis 24 h post-injury (Fig. 6c). Also in this setting, monocytes acquired an anti-inflammatory phenotype upon co-culture with EGC supernatant with increased expression of *Arg1* and decreased expression of *il12* similar to that seen in bone marrow monocytes (Fig. 6d and Supplementary Fig. 6e). Furthermore, flow cytometric analysis of bone marrow monocytes stimulated with EGC supernatant confirmed the increased expression of CD206 at protein level, while CCR2 expression was reduced, along with increased survival compared to control monocytes (Fig. 6e–h). These results were further underscored by the ability of EGC secreted factors to induce the expression of CD206 on sorted Ly6C^+^ monocytes from the muscularis 24 h post-injury and decrease the expression of CCR2 (Fig. 6i). Taken together, our ex vivo data suggest a direct interaction between EGCs and monocytes that could support the resolution of inflammation.

As cellular interaction analysis by NicheNet identified Csf1-Csf1r as the top candidate for potential ligand-receptor interaction between EGCs and monocytes (Fig. 5f), we next aimed to determine whether EGC-derived CSF1 had the potential to induce anti-inflammatory CD206^+^ Mφs in our ex vivo co-culture model by antibody-mediated CSF1r blockade. Anti-CSF1r treatment in combination with EGC supernatant attenuated the differentiation of both bone marrow and muscularis monocytes into anti-inflammatory Mφs with reduced CD206 expression, while increased CCR2 expression was only observed for bone marrow monocytes compared to cells stimulated with EGC supernatant alone (Fig. 6e–i). No effect was observed on survival, CCR2 and CD206 expression when bone marrow monocytes were treated with anti-CSF1r compared to control monocytes, while stimulation of bone marrow monocytes with CSF-1 increased survival and reduced CCR2 expression with no effect on CD206 expression (Supplementary Fig. 6f–h). These results underscore that CSF-1 together with other factors are crucial for the differentiation of monocytes into anti-inflammatory CD206^+^ Mφs. Altogether, these data provide evidence for a direct interaction between monocytes and EGC-derived ligands ex vivo which stimulate the differentiation into anti-inflammatory CD206^+^ Mφs.

### EGCs are involved in the differentiation of monocytes into CD206^±^ Mφs, which are able to limit damage to EGCs through CSF-1 signaling during muscularis inflammation

**Fig. 7:**
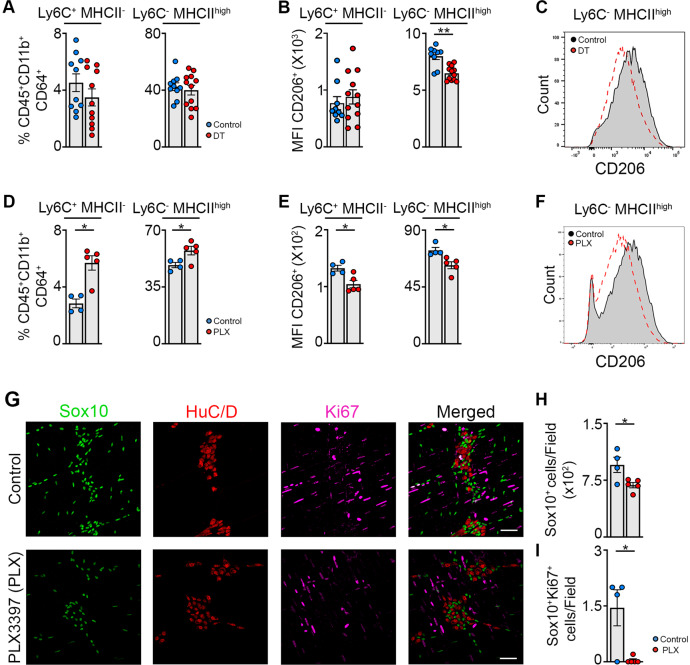
EGCs are crucial for monocyte differentiation into anti-inflammatory CD206^+^ Mφs in part via CSF-1 in vivo. **a**–**c** A small portion of the small intestine of PLP^CreERT2^ iDTR mice was exposed to saline or DT after intestinal manipulation and mice were sacrificed 72 h after the induction of muscularis inflammation. **a** Percentages of Ly6C^+^ MHCII^−^ monocytes and Ly6C^−^ MHCII^hi^ Mφs in live CD45^+^ CD11b^+^ Ly6G^−^ CD64^+^ population. **b** Mean fluorescent intensity (MFI) of CD206 in Ly6C^+^ MHCII^−^ monocytes and Ly6C^−^ MHCII^hi^ Mφs. **c** Histogram of CD206 expression in Ly6C^−^ MHCII^hi^ Mφs in control and DT exposed PLP^CreERT2^ iDTR mice 72 h after muscularis inflammation. **d**–**h** Wild-type mice were gavaged daily with vehicle or PLX-3397 (50 mg/kg) starting from the day of the manipulation and sacrificed 72 h after the induction of muscularis inflammation. **D** Percentages of Ly6C^+^ MHCII^−^ monocytes and Ly6C^−^ MHCII^hi^ Mφs in live CD45^+^ CD11b^+^ Ly6G^−^ CD64^+^ population. **e** Mean fluorescent intensity (MFI) of CD206 in Ly6C^+^ MHCII^−^ monocytes and Ly6C^−^ MHCII^hi^ Mφs. **f** Histogram of CD206 expression in Ly6C^−^ MHCII^hi^ Mφs in control and PLX treated mice 72 h after muscularis inflammation. **g** Immunofluorescent images of muscularis whole-mounts in control and PLX treated mice 72 h after muscularis inflammation stained for SOX10 (green), HuC/D (red) and Ki-67 (purple). Scale bar 25 µm. **h** Quantification of the number of SOX10^+^ cells per field in control and PLX treated mice 72 h after muscularis inflammation (average of 4–5 pictures/mouse). **i** Quantification of the number of Ki-67^+^ SOX10^+^ cells per field in control and PLX treated mice 72 h after muscularis inflammation (*N* = 4–5 pictures/mouse). **a**, **b**; **d**, **e**; **h**, **i**. *T*-test. *p < 0.05; **p < 0.01.

In order to definitively determine the contribution of EGCs in the differentiation of monocytes into anti-inflammatory CD206^+^ Mφs, 8-week-old PLP^CreERT2/+^ iDTR (PLP-iDTR) mice were injected with tamoxifen and a small part of the small intestine was exposed to saline or diphteria toxin (DT) at 12 weeks of age following the induction of muscularis inflammation. 72 h post-injury, there was a significant reduction of the volume and intensity of GFAP (Supplementary Fig. 7a, b) as well as in the number of Sox10^+^ EGCs (Supplementary Fig. 7a, c) in the DT treated PLP-iDTR mice compared to the control animals, thereby confirming EGC depletion upon DT administration. While no differences were observed in the percentage and cell numbers of the myeloid populations 72 h after the induction of muscularis inflammation (Fig. 7a and Supplementary Fig. 7d), there was a significant reduction of the MFI of CD206 in Ly6C^−^ MHCII^hi^ Mφs of PLP-iDTR mice treated with DT compared with their control (Fig. 7b, c). These results are in line with our scRNA-seq data, where the highest CD206 expression was observed in the Mφ subpopulation that expresses high levels of MHCII genes. Therefore, we can conclude that EGCs are able to stimulate the differentiation of monocytes into CD206^+^ MHCII^hi^ Mφs in vivo during muscularis inflammation.

Based on the evidence that EGC-derived CSF-1 is able to stimulate the differentiation of monocytes into CD206^+^ Mφs with an anti-inflammatory phenotype in vitro, we hypothesized that blocking monocyte differentiation towards anti-inflammatory CD206^+^ Mφs in vivo via CSF1R inhibition might have neurodegenerative effects on EGCs. To test this hypothesis, WT mice were treated daily with pexidartinib (PLX-3397), an oral tyrosine kinase inhibitor of CSF1R, from the day of the induction of muscularis inflammation until the sacrifice at 72 h post-injury. Although long-term CSF1R inhibition can lead to Mφ depletion in vivo^30,31^, short-term PLX-3397 treatment did not result in an overall reduction of differentiated Mφs. Rather, we observed an increased percentage and cell number of neutrophils, monocytes and MHCII^hi^ Mφs compared to control treated animals 72 h post-injury (Fig. 7d and Supplementary Fig. 7e, f), showing normal development of the Mφ compartment with increased muscularis inflammation. These results can be explained by a reduced expression of CD206 in both monocytes and MHCII^hi^ Mφs in PLX treated mice compared to control mice (Fig. 7e, f), indicating a more pro-inflammatory phenotype of these myeloid subsets. As anti-inflammatory Mφs are essential to support the neuronal compartment in the muscularis externa^7^, we next sought to evaluate the effect of diminished CD206 expression on MHCII^hi^ Mφs on EGC growth and survival upon PLX treatment. Based on the immunofluorescent quantification of Sox10^+^ and Sox10^+^ Ki-67^+^ EGCs, we could observe that there was a reduction of Sox10^+^ EGCs in the PLX treated mice as compared to the control mice. In addition, in WT mice there was a small subset of EGCs that was Ki-67^+^ 72 h post-injury which was significantly reduced upon PLX treatment (Fig. 7g–i). A similar reduction in the number of Sox10^+^ EGCs was also observed in CCR2^−/−^ mice as compared to WT mice 72 h after the induction of muscularis inflammation (Supplementary Fig. 7h, i). Based on these results, we conclude that EGCs are involved in the differentiation of monocytes into CD206^+^ Mφs in vivo, limiting damage to the EGCs during muscularis inflammation.

## Discussion

Previous studies have shown that monocyte-derived Mφs are essential to resolve inflammation and restore gut motility following muscularis inflammation^11,21^. To date, it remains to be determined which Mφ subpopulations are responsible for tissue recovery following muscularis inflammation and which factors induce the pro-resolving Mφ phenotype. Our work provides a comprehensive overview of the differentiation of incoming monocytes into pro-resolving Mφs during muscularis inflammation, thereby characterizing gene expression dynamics during monocyte-to-Mφ transitions and uncovering transcriptional regulators of different Mφ subpopulations. Furthermore, we have identified a role for EGCs during muscularis injury, which via increased production of the monocyte chemoattractant CCL2 and elevated levels of multiple mediators could be involved in the differentiation of monocytes towards anti-inflammatory Mφs, thereby promoting tissue recovery.

Using time-resolved scRNA-seq of immune cells isolated from the inflamed muscularis, we revealed the heterogeneity and differentiation trajectory of different monocyte and Mφ subpopulations at homeostasis and during early and late inflammation. During acute muscularis inflammation, Ly6c^+^ monocytes were present along with other transient monocyte and Mφ subpopulations such as Fabp5^+^ and Arg1^+^ Mo/Mφs, showing substantial heterogeneity in monocytic cells during acute inflammation, rather than a single homogenous wave of Ly6c^+^ monocytes. Additionally, we identified Ccr2^+^ int Mφs as a transitional state between Ly6c^+^ monocytes and Cd206^+^ Mφs. These intermediate cells were mainly present near the branching point or in the branch of the trajectory diverging towards Cd206^+^ Mφs and resembled Ly6C^+^ MHCII^+^ immature Mφs characterized by flow cytometry. Furthermore, we identified two main pro-resolving Mφ subpopulations during the resolution of muscularis inflammation characterized by Cd206^+^ MhcII^hi^ and Timp2^+^ MhcII^lo^ gene signatures. Trajectory interference analysis during muscularis inflammation showed that these subpopulations are derived from infiltrating Ly6c^+^ monocytes. In line, scRNA-seq analysis showed that these two myeloid subpopulations are absent from the inflamed muscularis of CCR2 deficient mice, further supporting their monocytic origin. Accordingly, flow cytometric analysis of CX3CR1^GFP/+^ mice during muscularis inflammation showed that CX3CR1^lo^ MHCII^hi^ and CX3CR1^lo^ MHCII^lo^ Mφs are derived from CCR2^+^ monocytes and resemble Cd206^+^ and Timp2^+^ Mφs, respectively. Both phenotypically and functionally, Cd206^+^ MhcII^hi^ Mφs showed high resemblance to homeostatic Cx3cr1^+^ Mφs with a similar gene signature and activity of specific regulons (e.g., *Junb, Irf8)*^32,33^, while Timp2^+^ MhcII^lo^ Mφs displayed a different gene signature and regulon activity (e.g., *Cebpb, Gata6)*^34,35^. In this regard, Cd206^+^ Mφs were shown to have high regulon activity of *Irf8*, a transcription factor involved in the regulation of genes important for antigen-presentation (*Cd86* and *Cd40*) and for lymphoid and myeloid chemotaxis (*Cxcl9, Cxcl10, Ccl4, Ccl8*, and *Ccl12*)^33^. In addition, these cells upregulated genes involved in efferocytosis (*Axl* and *MerTK*) and exhibited high *MhcII* gene expression. These data suggest that CD206^+^ Mφs might respond to apoptotic cells in their vicinity and undergo differentiation into an anti-inflammatory phenotype in response to efferocytosis. On the other hand, Timp2^+^ Mφs have high regulon activity of *Cebpb*, which typically controls the expression of anti-inflammatory genes such as *Msr1, Il10, II13ra*, and *Arg1*. Further studies on small and large peritoneal Mφs could be instrumental in understanding Mφ biology during muscularis inflammation, as these cells resemble Cd206^+^ and Timp2^+^ Mφs respectively, based on gene expression and regulon activity^36,37^. Furthermore, we also found other subpopulations that were characterized by highly specific gene signatures and regulon activities, such as Fabp5^+^ Mφs with enhanced *Nr1h3 (LXRα)* and *Pparγ* regulon activity, which may be involved in iron handling (*Flt1, Fth1, Hmox1*) and detoxification (*Mt1, Akr1a1, Prdx1, Cd36*). Further studies will be essential to unravel the functional roles of these different Mφ subsets during muscularis inflammation by defining their precise tissue localization and assessing their contribution to the pathophysiological response during local tissue damage.

The muscularis micro-environment has been shown to be highly regulated by a network of enteric neurons and EGCs constituting the ENS. Here, Mφs play an essential role to support neuronal function via the secretion of BMP-2^1,6^, while neurons in turn produce CSF1, vital for Mφ maintenance^6^. In the current study and in line with Grubisic et al., we have shown that CSF1 can also be produced by EGCs^19^. This close neuro-immune interaction between the ENS and Mφs at homeostasis and during disease resembles the one found in other neuronal tissue niches. For instance, in the central nervous system, there is extensive crosstalk between astrocytes and microglia, critical for brain homeostasis^38^. Upon brain injury, microglia are the first cells to be recruited to the site of damage to phagocytose dead cells and debris^39^, similar to what is observed during muscularis inflammation^11,21^. Concurrently, reactive astrocytes are activated to release pro-inflammatory mediators that affect microglia and can recruit different leukocytes. Subsequently, astrocytes increase their expression of GFAP and undergo morphological changes leading to the formation of glial scars^40^. A recent report by Liddelow et al. proposed astrocyte polarization into A1 and A2 states as a response to various stimuli^27^. In line with our findings, they showed that IL-1β and IL-1α induce the expression of most pan-reactive marker genes and the A2 marker genes along with a minority of A1 markers including Gfap. Similarly, we have shown a comparable process taking place at the level of the ENS, in which EGCs are activated during the early stages of intestinal inflammation by damage to the micro-environment. In line with previous work from Stoffels et al.  ^41^, our GSEA analysis confirmed that early activation of EGCs during muscularis inflammation is dependent upon IL-1 cytokines. Furthermore, these damage-activated EGCs closely interact with Mφs in the muscularis and act as safeguards of the micro-environment by initiating an inflammatory response via CCL2 to protect the tissue from further damage. As opposed to the detrimental effects of astrocytes in brain injury, EGCs induce monocyte differentiation towards anti-inflammatory CD206^+^ Mφs by the production of cytokines and growth factors, including CSF1. This process seems to be crucial for the resolution of ENS damage, as Mφs are able to stimulate EGC proliferation in vitro and depletion of EGCs in vivo leads to reduced expression of CD206 in MHCII^hi^ Mφs. Recently, it was also shown that during experimental autoimmune encephalomyelitis, astrocytes provide soluble signals to pro-inflammatory Mφs to induce an anti-inflammatory phenotype^42^. In line, blocking CSF1-CSF1R signaling (using an oral CSF1R inhibitor, PLX-3397) increased monocyte and neutrophil infiltration in the muscularis during the recovery of muscularis inflammation, and reduced CD206 expression in MHCII^hi^ Mφs associated with reduced number of EGCs as well as EGC proliferation. Overall, EGC-derived CSF1 represents a potent signal to initiate anti-inflammatory Mφ differentiation essential for tissue recovery and for avoiding excessive injury to the ENS during muscularis inflammation.

Overall, these insights may pave the way to novel therapeutic approaches aiming to prevent complications related to alterations of the ENS and reduced GI motility in patients affected by intestinal inflammatory disorders. Our findings on the impact of anti-inflammatory signals from EGCs on myeloid cells could be extrapolated beyond the context of the intestinal inflammation and may be expanded to several other acute and chronic inflammatory conditions and autoimmune diseases.

## Material & methods

### Mouse Model

Twelve week old female wild-type (WT; C57BL/6 J), CCR2 knockout (CCR2^−/−^)^43^, Cx3cr1^GFP/+ ^^44^, Cx3cr1^CreERT2 ^^45^, Rosa26-LSL-YFP^46^, PLP^CreERT2 ^^47^, Rpl22^HA ^^24^, PLP^CreERT2 ^^47^, iDTR^48^ and their littermate controls were used. All lines were bred and maintained at the KU Leuven animal facility on a 12:12-h light-dark cycle and had *ad libitum* access to tap water and commercially available chow (ssniff^®^ R/M-H, ssniff Spezialdiäten GmbH). All experimental procedures were approved by the Animal Care and Animal Experiments Ethical Committee of KU Leuven.

### Experimental model of small intestinal inflammation

An established model of intestinal manipulation was used to induce small intestinal muscularis inflammation as previously reported^49^. In brief, animals were anesthetized by intraperitoneal injection of ketamine (Ketalar 100 mg/kg; Pfizer) and xylazine (Rompun 10 mg/kg; Bayer). After a midline laparotomy, the cecum and the small intestine were carefully externalized avoiding the pancreas and the ileocecal valve and stretching of the colon or the stomach. Next, the entire length of the exteriorized small intestine was manipulated three times back and forth using a purpose-designed device that enables the application of a constant pressure to the intestine by a cotton swab applicator attached to its end.

### Statistical analysis

Results are shown as mean ± standard error of the mean (SEM). Significance between two mean groups was determined by an unpaired two-tailed Student’s *t*-test or a non-parametric Mann–Whitney test, while one-way analysis of variance (one-way ANOVA) followed by Dunnett’s Multiple comparison test was performed to compare the mean of multiple groups. GraphPad Prism V.9.1.0 software (GraphPad Inc) was used to generate graphs and to perform statistical analysis.

## Supplementary information

Supplementary Information

## Data Availability

Single cell RNA sequencing and bulk RNA sequencing data generated for this study are available through Gene Expression Omnibus with accession number GSE167465.
