# Supplementary material for: Enteric glial cells favor accumulation of anti-inflammatory macrophages during the resolution of muscularis inflammation
Source: Mucosal Immunol. 2022 Sep 7;15(6):1296–308. doi: 10.1038/s41385-022-00563-2 (PMC9705256; doi:10.1038/s41385-022-00563-2)
Supplement: Supplementary file 1 — Supplementary Information [file 41385_2022_563_MOESM1_ESM.pdf]

Supplementary information:

Enteric glial cells favour accumulation of anti-inflammatory macrophages during the resolution of muscularis inflammation

Short title: EGC-Mφ interaction in muscularis inflammation

Michelle Stakenborg[1, #], **Saeed Abdurahiman**[1, #], Veronica De Simone[1], Gera Goverse[1], Nathalie Stakenborg[1], Lies van Baarle[1], Qin Wu[1], Dimitri Pirottin[2], Jung-Seok Kim[3], Louise Chappell-Maor[3], Isabel Pintelon[4], Sofie Thys[4], Emilie Pollenus[5], Louis Boon[6], Philippe Van den Steen[5], Marlene Hao[1], Jo A. Van Ginderachter[7,8], Guy E. Boeckxstaens[1], Jean-Pierre Timmermans[4], Steffen Jung[3], Thomas Marichal[9,10], Sales Ibiza[1,4,*], Gianluca Matteoli[1,*]

Affiliation
[1]Department of Chronic Diseases, Metabolism and Ageing (CHROMETA), Translational Research Center for Gastrointestinal Disorders (TARGID), KU Leuven, Leuven, Belgium.
[2]Laboratory of Cellular and Molecular Immunology, GIGA Institute, Liege University, Liege, Belgium.
[3]Department of Immunology, Weizmann Institute of Science, Rehovot, Israel.
[4]Laboratory of Cell Biology & Histology, Department of Veterinary Sciences, University of Antwerp, Antwerp, Belgium.
[5]Laboratory of Immunoparasitology, Department of Microbiology, Immunology and Transplantation, Rega Institute for Medical research, KU Leuven, Leuven, Belgium
[6]Polpharma Biologics, Utrecht, the Netherlands.
[7]Cellular and Molecular Immunology Lab, Department of Bio-engineering Sciences, Vrije Universiteit Brussel, Brussels, Belgium.
[8]Myeloid Cell Immunology Lab, VIB Center for Inflammation Research, Brussels, Belgium.
[9]Laboratory of Immunophysiology, GIGA Institute, Liege University, Liege, Belgium.
[10]Department of Functional Sciences, Faculty of Veterinary Medicine, Liege University, Liege, Belgium.

#, these authors contributed equally to this work.
*, senior authors: Sales Ibiza and Gianluca Matteoli

Corresponding author:
Prof. Sales Ibiza, PhD
Laboratory of Cell Biology & Histology
Department of Veterinary Sciences University of Antwerp,
Campus Drie Eiken, Universiteitsplein 1, B-2610 Wilrijk, Belgium
T +32-3-2653324
Email: Sales.IbizaMartinez@uantwerpen.be

Prof. Gianluca Matteoli, DVM, PhD
Laboratory of Mucosal Immunology
Department of Chronic Diseases, Metabolism and Ageing (CHROMETA)
KU Leuven, Herestraat 49, O&N1 box 701| BE-3000 Leuven | Belgium
Tel. + 32 (0)16 377566
Email: gianluca.matteoli@kuleuven.be

Supplementary methods:

Targeted ablation of enteric glial cells in the small intestine. To specifically eliminate enteric glial cells in the small intestinal, $PLP^{CreERT2}$ iDTR mice were injected twice with tamoxifen (2 mg; Sigma) dissolved in MIGLYOL®812 (Caesar & Loretz) to induce the expression of the diphteria toxin receptor 4 weeks prior to the induction of muscularis inflammation. Next, mice were manipulated using the standard protocol as previously reported[1] and the small intestine was re-inserted into the abdomen to avoid drying. The ileum and the last part of the jejenum were exteriorized and an area of 8 cm of small intestine was placed inside of a Petri dish filled with 500 µl of DT solution (4000 ng/ml; Sigma) and exposed for 10 min. Afterwards, the small intestine was dried with gauzed and placed back into the abdomen.

Administration of PLX-3397. PLX-3397 was purchased from Selleckchem Bio (S7818) and dissolved according to manufacturer's instructions in 5% DMSO, 45% PEG-300, 5% Tween-80 and 45% water. Mice were gavaged with 100 µl of vehicle or PLX-3397 at a concentration of 50 mg/kg 1 hour prior to intestinal manipulation. In addition, mice were gavaged daily 24h and 48h after the induction of intestinal inflammation.

Isolation and digestion of the muscularis. The small intestine was removed and flushed with ice-cold PBS. The muscularis was carefully removed with a forceps, cut in small pieces with scissors and digested in 2 mg/mL collagenase type IV (Gibco) in RPMI-1640 (Lonza) supplemented with 2% HEPES (Gibco), 2% FBS and 5U/mL DNase I (Roche) for 30 min at 37°C with continuous stirring. The resulting cell suspension was blocked using FACS buffer and passed through a 70 µm cell strainer (BD Falcon), after which cells were spun down at 400 x g for 8 min at 4°C. Cells were counted using the Countess II FL (Thermo Fisher).

Immunofluorescence. The small intestine was removed and flushed with ice-cold PBS to remove luminal contents. Then, the intestine was cut open longitudinally before stretching and was fixed for 30 min in 4% PFA at room temperature. Next, the tissue was washed three times with PBS and the mucosa and submucosa were removed with a forceps to obtain whole-mounts of the muscularis, after which it was permeabilized and blocked in 0.3% Triton X-100

and 3% BSA in PBS for 2h at room temperature. Subsequently, samples were incubated for 48h at 4°C with the following primary antibodies: chicken anti-neurofilament (Abcam; ab72996), rat anti-GFP (Nacalai Tesque; GF090R), goat anti-CCL2 (R&D Systems; AF-479-NA), rabbit anti-GFAP (Dako; Z0334), chicken anti-GFAP (Abcam; ab4674), goat anti-TIMP2 (R&D systems, AF971), rabbit anti-CD206 (Abcam, ab64693), rabbit anti-HA (CST; Rb#3724), goat anti-SOX10 (R&D systems, AF2864), rabbit anti-Ki-67 (Abcam, ab15580), human ANNA-1 serum anti-HuC/D (kindly provided by Prof. Lennon V. A. (Mayo Clinic, Rochester, Minnesota, USA)[2]. Afterwards, samples were washed in PBS and incubated with DAPI (4′,6-Diamidine-2′-phenylindole dihydrochloride; Sigma-Aldrich) combined with the secondary antibodies: donkey anti-chicken Cy5 (Jackson), donkey anti-rat Alexa Fluor 488 (Invitrogen), donkey anti-rabbit Cy3 (Jackson), donkey anti-human Cy5 (Jackson), donkey anti-goat Cy3 (Jackson), donkey anti-rabbit Alexa Fluor 488 (Jackson) and donkey anti-rabbit FITC. All primary and secondary antibodies were dissolved in 0.3% Triton X-100 and 3% BSA in PBS. Finally, samples were rinsed three times in PBS and mounted with SlowFade Diamond Antifade mountant (Invitrogen). Images were recorded on a LSM780 or LSM880 multiphoton microscope (Zeiss) and a 25x or 63x objective (Zeiss) was used. Identical settings were used for all conditions in one experiment. Images were processed and analyzed using ImageJ (NIH) or Imaris software (Bitplane).

Lineage Tracing of CX3CR1$^+$ Macrophages. To induce CreERT2 recombinase activity to trace CX3CR1$^+$ muscularis Mφs, Cx3cr1^{CreERT2} mice were crossed with Rosa26-LSL-YFP mice to obtain double heterozygous mice. Mice aged 8 weeks were injected three times subcutaneously with 4 mg TAM (Sigma-Aldrich, St. Louis, USA) per 30 g body weight dissolved in corn oil (Sigma-Aldrich) every other day as previously described[3]. Mice were sacrificed 4 weeks after the first tamoxifen injection.

Flow Cytometry and Sorting of Live Cells. Single cell suspensions were blocked with rat anti-mouse CD16/CD32 (Fc block, BD Biosciences) for 10 min and afterward incubated with fluorophore-conjugated anti-mouse antibodies at recommended dilutions for 20 min at 4°C.

Dead cells were excluded using 7-AAD (BD Biosciences) or Fixable Viability Dye eFluor 450 (eBiosciences). During FACS acquisition, doublets were excluded. FACS antibodies used can be found in **Table 1**. Samples were acquired using a Symphony (BD Biosciences) and analyzed with FlowJo software (version 4.6.2, Treestar). For cell sorting, a BD Aria III (BD Biosciences) was used.

Anti	Conjugate	Company	Cat. No.	Clone
CD45	APC-eFluor 780	eBioscience	47-0451-82	30-F11
CD45	BV510	Biolegend	103138	30-F11
CD45	FITC	BD Pharmingen	553772	104
CD11b	PE-Cy7	BD Pharmingen	552850	M1/70
Ly6C	BV421	BD Pharmingen	562727	AL-21
Ly6C	PE	BD Pharmingen	560592	AL-21
CD64a and b	BV711	Biolegend	139311	X54-5/7.1
CD64a and b	BV421	Biolegend	139309	X54-5/7.1
CD206	Alexa Fluor 647	BioLegend	141712	C068C2
CCR2	BUV395	BD Pharmingen	747972	475301
Ly6G	BUV563	BD Pharmingen	612921	IA8
Ly6G	APC	BD Pharmingen	560599	IA8
MHCII (I-A/I-E)	APC-eFluor 780	eBioscience	47-5321-82	M5/114.15.2

Table 1: FACS antibodies

Muscularis ganglia isolation from adult mice. The small intestine was flushed with cold bicarbonate/CO_2 KREBS buffer. Thereafter, the myenteric plexus was collected by removing the mucosa and submucosa as previously described[4]. The extracted tissue was subsequently placed in GentleMACS™ tubes (Miltenyi Biotec, Germany) with 5mL of pre-warmed dissociation medium DMEM/F12 (Lonza, Belgium) supplemented with 10% FBS (Biowest, USA), 100U/mL Penicillin/Streptomycin, 2mM L-glutamine (Lonza, Belgium), 45mg/mL NaHCO3, 0.01 mg/mL Gentamicin and 0.25 µg/mL Amphotericin B (Thermo Fisher). Additionally, 400µl BSA (50g/L), 250µl protease (20mg/mL) and 250µl collagenase type IV (20mg/mL) (Sigma-Aldrich, Belgium) were added for enzymatic dissociation of tissue. The GentleMACs™ tube was then placed into the GentleMACS™ Dissociator (Miltenyi Biotec, Germany) for mechanical dissociation for 2 min at room temperature. The tube was subsequently placed in a water bath at 37°C for 13 min with shaking. Subsequently, a second mechanical dissociation was performed, followed by stopping the enzymatic dissociation by addition of cold KREBS buffer. Samples were spun down at 407 x g at 4°C and re-suspended

in dissociation medium without enzymes, plated in a petri dish and placed under an inverted binocular microscope, where the ganglia were picked up with a pipette tip and were lysed with RLT buffer for assessment of RNA expression levels.

Neurosphere-derived embryonic enteric glial cell culture. Neurosphere-derived EGCs were obtained as previously described[5]. Briefly, total intestines from E14.5 C57BL/6J mice were digested with collagenase D (0.5 mg/mL; Roche) and DNase I (0.1mg/mL; Roche) in DMEM/F-12, GlutaMAX, supplemented with 1% HEPES, streptomycin/penicillin and 0.1% β-mercaptoethanol (Gibco) for approximately 30 min at 37°C under gentle agitation. Cells were cultured for 1 week in a CO_2 incubator at 37°C in DMEM/F-12, GlutaMAX, streptomycin and penicillin and 0.1% β-mercaptoethanol (Gibco) supplemented with B27 (Gibco), 40 ng/mL EGF (Gibco) and 20 ng/mL FGF (Gibco). After 1 week of culture, neurospheres were treated with 0.05% trypsin (Gibco), transferred into PDL (Sigma-Aldrich) coated plates and differentiated in DMEM supplemented with 10% FBS, 1% HEPES, glutamine, streptomycin and penicillin and 0.1% β-mercaptoethanol (Gibco) until confluence for 5 days to obtain EGC supernatant. In addition, neurosphere-derived EGCs were stimulated with IL1-α (Peprotech) and/or IL1-β (Peprotech) for 18 hours.

RNA sequencing of neurosphere-derived EGC. RNA was isolated using RNeasy Micro Kit according to the manufacturer's instructions. A cDNA indexed library was prepared using the Illumina TruSeq RNA library Kit and sequencing was performed on the Illumina NextSeq500 platform at the Nucleomics core (VIB, Leuven). The reads were preprocessed by trimming for quality and to remove adapters (cutadapt 1.15 and FastX 0.0.14) followed by filtering for quality (FastX 0.0.14 and ShortRead 1.40.0) and removal of contaminants (bowtie 2.3.3.1). The reads were then mapped against the reference genome GRCm3882 using STAR (2.5.2b). Further filtering, sorting and indexing was done using samtools 1.5 and counts file was prepared and differential expression analysis was performed using EdgeR.

Monocyte isolation. Mouse bone marrow monocytes were isolated from WT mice. Briefly, the tibia and femur of mice were dissected. Bone marrow cells were flushed with DMEM high

glucose (Lonza) supplemented with 10% FBS. After cells were collected and counted, monocytes were isolated with the EasySep™ Mouse Monocyte Isolation Kit (Stem Cell Technologies, Vancouver, Canada) according to the manufacturer's instructions. Next, 10^5 monocytes were stimulated for 24-48h with EGC supernatant, CSF-1 (50 ng/mL; PeproTech) and/or anti-CSF1r (5µg/mL; BE0213; BioXCell) at 37°C. Cells were stimulated with LPS (100 ng/ml; Sigma-Aldrich) 6 hours prior to the end of the experiment. Monocytes were analyzed by flow cytometry or lysed with RLT buffer.

EGC proliferation assay. After 1 week of culture, enteric neurospheres were collected and dissociated to generate a single-cell suspension by using the NeuroCult™ Chemical Dissociation Kit (Mouse) (Stem Cell Technologies, CatN°05707). Next, cells were filtered through a 70µm cell strainer and counted using a burker chamber. EGCs were transferred into poly-D-Lysine (Gibco, CatN°A3890401) coated 96-well plates at a concentration of 5000 cells per well and 100 µl of conditioned medium was added to each well. To obtain conditioned medium from myeloid cells, $Ly6C^{hi}$ monocytes from 24h post-injury and $Ly6C^{-}$ $MHCII^{hi}$ Mφs from 72h post-injury were sorted from the muscularis and cultured at a concentration of 50.000 cells/well per 400µl medium with 0.5% FBS during 24h. After 5 days of culture in (un)conditioned media, the proliferation of the EGCs was measured by using the Cell Counting Kit-8 (Tebu-bio, CatN°CK04-05). After 2 hours of incubation with the CCK-8 solution, the absorbance was measured at 450nm and 600nm (background signal) using a microplate reader. Data were expressed as induction with respect to EGCs cultured with unconditioned medium.

RNA extraction and gene expression of ganglia and myeloid cells. Picked ganglia from the small intestine and monocytes/macropahges were lysed in RLT buffer and stored at -80°C. RNA extraction was performed using RNeasy Mini Kit for tissue and high cell numbers (Qiagen) or RNeasy Micro Kit for low cell numbers following manufacturer's instructions. Total RNA was transcribed into cDNA by the High-Capacity cDNA Reverse Transcription Kit (Thermo Fisher) according to manufacturer's instructions. Quantitative real-time transcription

polymerase chain reactions (RT-PCR) were performed with the LightCycler 480 SYBR Green I Master (Roche) on the Light Cycler 480 (Roche). Results were quantified using the 2-ΔΔCT method[6]. Expression levels of the genes of interest were normalized to the expression levels of the reference gene Rpl32. Results are expressed as mean values and primer sequences used are listed in **Table 2**.

	Forward primer	Reverse primer
Rpl32	AAGCGAAACTGGCGGAAAC	TAACCGATGTTGGGCATCAG
Arg1	CAGAAGAATGGAAGAGTCAG	CAGATATGCAGGGAGTCACC
Ccl2	CAGGTGTCCCAAAGAAGCTGTA	CATTTGGTTCCGATCCAGG
Csf1	ATGAGCAGGAGTATTGCCAAGG	TCCATTCCCAATCATGTGGCTA
Il10	CCAAGCCTTATCGGAAATGA	TCACTCTTCACCTGCTCCAC
Il6	CCATAGCTACCTGGAGTACATG	TGGAAATTGGGGTAGGAAGGAC
Il12	CCTGCTGAAGACCACAGATG	AGCTCCCTCTTGTTGTGGAA
Mrc1	CTCTGTTCAGCTATTGGACGC	TGGCACTCCCAAACATAATTTGA
Nos2	GCTTCTGGTCGATGTCATGAG	TCCACCAGGAGATGTTGAAC

Table 2: List of RT-PCR primers

Isolation and sequencing of ribosome-associated mRNA from EGCs. To induce gene recombination of $Rpl22^{HA}$ in $PLP1^{CreERT2}$ $Rpl22^{HA}$ transgenic mice at 10-12 weeks of age, mice were injected twice intraperitoneally with 100 µl of 15 mg/mL tamoxifen (Sigma-Aldrich) dissolved in Miglyol 812 (Caesar & Loretz, Hilden, Germany). All animals were sacrificed 3-4 weeks after the first injection. The whole small intestine of each mouse was peeled by removing the mucosa and submucosa and samples were flash-frozen in liquid nitrogen and stored at -80°C until further processing. Samples were immunoprecipitated at the Weizman Instiute (Israel) according to the published protocol[7, 8]. In brief, samples were homogenized in 4 mL cold homogenization buffer (50 mM Tris, pH 7.4, 100 mM KCl, 12 mM MgCl2, 1% NP-40, 1 mM DTT, 1:100 protease inhibitor (Sigma-Aldrich), 200 U/mL RNasin (Promega) and 0.1 mg/mL cycloheximide (Sigma-Aldrich)) with a Dounce homogenizer (Sigma-Aldrich) until the suspension was homogeneous. To remove cell debris, 2 mL of the homogenate was transferred to a microcentrifuge tube and centrifuged at 10,000 g at 4°C for 10 min. Supernatants were transferred and split, and 8 µL (10 µg) of anti-HA antibody (H9658, Sigma-Aldrich) or 20 µL (10 µg) of mouse monoclonal IgG1 antibody (Sigma-Aldrich, Cat# M5284)

was added to the supernatant, followed by 4h of incubation with slow rotation at 4°C. Meanwhile, Dynabeads Protein G (Thermo Fisher), were equilibrated to homogenization buffer by washing three times. At the end of 4 h of incubation with antibody, beads were added to each sample, followed by incubation overnight at 4°C. Next, samples were washed three times with high-salt buffer (50 mM Tris, 300 mM KCl, 12 mM MgCl2, 1% NP-40, 1 mM DTT, 1:200 protease inhibitor, 100 U/mL RNasin and 0.1 mg/mL cycloheximide). At the end of the washes, beads were magnetized and excess buffer was removed, 150 µl lysis buffer was added to the beads and RNA was extracted with Dynabeads mRNA Direct purification kit (Thermo Fisher) according to manufacturer's instructions. RNA was eluted in 6 µl H_2O and used for RNA-seq.

RNAseq library preparation and analysis of immunoprecipitated samples. Immunoprecipitated samples were processed and analyzed according to published protocols[7, 8]. In brief, mRNA was captured with Dynabeads oligo(dT) (Life Technologies) according to manufacturer's guidelines. A bulk variation of MARSseq[9] was used to prepare libraries for RNA-seq. RNA was reversed transcribed with MARSseq barcoded RT primer in a 10 mL volume with the Affinity Script kit (Agilent). Reverse transcription was analyzed by qRT-PCR and samples with a similar CT were pooled (up to eight samples per pool). Each pool was treated with Exonuclease I (NEB) for 30 min at 37°C and subsequently cleaned by 1.2 x volumes of SPRI beads (Beckman Coulter). Subsequently, the cDNA was converted to double-stranded DNA with a second strand synthesis kit (NEB) in a 20mL reaction, incubating for 2 h at 16°C. The product was purified with 1.4 x volumes of SPRI beads, eluted in 8 mL and in vitro transcribed (with the beads) at 37°C overnight for linear amplification using the T7 High Yield RNA polymerase in vitro transcription kit (NEB). Following in vitro transcription, the DNA template was removed with Turbo DNase I (Ambion) for 15 min at 37°C and the amplified RNA (aRNA) was purified with 1.2 volumes of SPRI beads. The aRNA was fragmented by incubating in Zn^{2+} RNA fragmentation reagents (Ambion) for 3 min at 70°C and purified with 2 x volumes of SPRI beads. The aRNA was ligated to the MARS-seq ligation adaptor with T4 RNA Ligase I (NEB). The reaction was incubated for 2 h at 22°C. After 1.5 x SPRI cleanup, the ligated

product was reverse transcribed using Affinity Script RT enzyme (Agilent) and a primer complementary to the ligated adaptor. The reaction was incubated for 2 min at 42°C, for 45 min at 50°C, and for 5 min at 85°C. The cDNA was purified with 1.5 x volumes of SPRI beads. The library was completed and amplified through a nested PCR reaction with 0.5mM of P5_Rd1 and P7_Rd2 primers and PCR ready mix (Kappa Biosystems). The amplified pooled library was purified with 0.7 x volumes of SPRI beads to remove primer leftovers. Library concentration was measured with a Qubit fluorometer (Life Technologies) and mean molecule size was determined with a 2200 TapeStation instrument. RNaseq libraries were sequenced using the Illumina NextSeq 2000.

Data analysis was performed by using the UTAP transcriptome analysis pipeline[10]. Raw reads were trimmed using cutadapt with the parameters: -a AGATCGGAAGAGCACACGTCTGAACTCCAGTCAC -a "A–times 2 -u 3 -u -3 -q 20 -m 25). Reads were mapped to the genome (mm10, Gencode annotation version 10.0) using STAR (v2.4.2a) with the parameters –alignEndsType EndToEnd, –outFilterMismatchNoverLmax 0.05, –twopassMode Basic, –alignSoftClipAtReferenceEnds No. The pipeline quantifies the 3' of Gencode annotated genes. UMI counting was done after marking duplicates (in-house script) using HTSeq-count in union mode. Only reads with unique mapping were considered for further analysis, and genes having minimum 10 reads in at least one sample were considered. Gene expression levels were calculated and normalized using DESeq2 (1.26.0) with default parameters except alpha = 0.05[11].

Single cell RNA sequencing – Library preparation and sequencing. Single cell RNA sequencing libraries were prepared using the 10X Genomics platform according to manufacturer's instructions (Single cell 3' solution) and sequencing was performed on Illumina NextSeq500 (Illumina). Cell Ranger software (v3.0.2; 10X Genomics) was used to demultiplex Illumina BCL files to FASTQ files (cellranger mkfastq) for alignment to mouse GRCm38/mm10 genome, filtering, UMI counting, and to produce gene-barcode matrices. Library preparation,

sequencing and sample demultiplexing were performed at the Genomics Platform of the GIGA Institute (Liège University, Belgium).

Single-cell RNA sequencing Clustering and Dimensionality reduction. The filtered count matrices obtained after pre-processing with Cellranger were concatenated to obtain a combined raw count matrix which was then analyzed using the Seurat R package (3.1.3). Cells with less than 200 genes, cells with more than 10% mitochondrial genes and genes with expression in less than 3 cells were discarded from the analysis. SCTransform function was used for normalization and scaling. A regression of the percentage of mitochondrial genes was performed during the SCTransform step. Next, principal component analysis was used to reduce the dimension of the data before UMAP construction and clustering. The cells annotated as monocytes and Mφs were then employed to subset original concatenated counts matrix. The subset matrix was then subjected to the same process of normalization, scaling (SCTransform), principal component analysis, UMAP projection and clustering. The MHC II signature score was calculated using 'AUCell' R package as the 'area under the curve' with the following genes - H2-Ab1, H2-Aa, H2-Eb1, H2-Eb2, H2-DMa, H2-DMb1, H2-DMb2. During the re-clustering of monocytes and Mφs from WT and CCR2$^{-/-}$ mice, 67 cells appeared to be granulocytes/DCs (based on *Cxcr2* and *Cd209a* expression) and were not included in the re-clustering analysis of mono/Mφs. Analysis plots were generated using the R packages ggplot (3.2.1), pheatmap (1.0.12) and EnhancedVolcano (1.4.0). Markers for different clusters were determined using a Wilcoxon rank test with FindMarkers and FindAllMarkers functions in Seurat. Non-default parameters used at each step during clustering or re-clustering with Seurat are given in **Table 3**.

scRNA seq data of Cd45+ sorted cells from Naïve mice/ 24h post IM/ 72 post IM as dpicted in Fig 1

**RunUMAP**	dims	umap.method	metric	n.neighbours	min.dist
	1 to 40	uwot	correlation	50L	0,5
**FindNeighbours**	dims	prune.snn	k.param		
	1 to 40	1/15	20		
**FindClusters**	resolution				
	0,5				

scRNA seq data of Cd45+ sorted cells from WT/CCR2 KO mice 24h post IM/ 72 post IM

**RunUMAP**	dims	umap.method	metric	n.neighbours	min.dist
	1 to 40	uwot	correlation	50L	0,5
**FindNeighbours**	dims	prune.snn	k.param		
	1 to 40	1/15	20		
**FindClusters**	resolution				
	0,5				

Reclustering of Monocytes and Macrophages cells from Fig1 as depicted in Fig 2

**RunUMAP**	dims	umap.method	
	1 to 30	uwot	
**FindNeighbours**	dims	prune.snn	k.param
	1 to 30	1/10	40
**FindClusters**	resolution		
	0,8		

Reclustering of Monocytes and Macrophages cells from WT/CCR2 KO mice data as depicted in Fig 4

**RunUMAP**	dims	umap.method	
	1 to 30	uwot	
**FindNeighbours**	dims	prune.snn	k.param
	1 to 30	1/10	40
**FindClusters**	resolution		
	0,7		

Supplementary Table 3: Non-default parameters Seurat.

Trajectory inference using Monocle-2. Monocle-2 was used with the raw count matrix of all monocytes and macrophages from WT data (depicted in Figure 2A). Cells were clustered with 'densityPeak' method (rho_threshold = 40, delta_threshold = 10). Top 3000 genes by q-value determined using differentialGeneTest() function were used as 'ordering genes'. Finally, DDRTree dimensionality reduction was performed and cells were ordered using orderCells() function.

Inferring cell-cell communication using NicheNet. We used NicheNet (nichenetr R package; version - 0.1.0) to study the interactions between EGCs and infiltrating monocytes after muscularis inflammation. To identify EGC derived ligands potentially inducing differentiation of Ly6c^{+} monocytes into Ccr2^{+} inte Mφs, RiboTag bulk sequencing data from PLP1^{CreERT2} Rpl22^{HA} mice in homeostasis and 3h after the induction of muscularis inflammation were used. Ligands were identified after filtering for genes upregulated 3h post-injury in PLP1^{+} EGCs with respect to homeostasis (adjusted p value < 0.05) and enriched in the HA sample with respect to the IgG control. Genes differentially expressed between Ccr2^{+} int Mφs and Ly6c^{+} monocytes (adjusted p value < 0.05) were considered as the gene set of interest. Genes expressed in at least 10% of the Ly6c+ monocyte cluster excluding genes in the gene set of interest were considered as the background expressed genes in Ly6c^{+} monocytes.

Single-cell regulatory network inference and clustering (SCENIC). SCENIC analysis was run as described by Aibar *et al.* (0.1.5) using the 10-thousand motifs annotation database and 500bp annotation database using pySCENIC (version 0.10.0)[12] on monocytes and Mφs (2249 cells). The input matrix was the UMI 'corrected', log normalized expression matrix. Databases of CisTarget - (mm10__refseq-r80__10kb_up_and_down_tss.mc9nr.feather, mm10__refseq-r80__500bp_up_and_100bp_down_tss.mc9nr.feather), and the transcription factor motif annotation database (motifs-v9-nr.mgi-m0.001-o0.0.tbl) from resources.aertslab.org/cistarget/, and the list of human transcription factors (mm_mgi_tfs.txt) from github.com/aertslab/pySCENIC/tree/master/resources were employed for the analysis. Significance of regulons for each cluster was assessed by a Wilcoxon test on AUC values.

Functional analysis of gene sets. Differentially upregulated genes in each cluster compared to the others were used with enrichGO function from the clusterprofiler R package (version 3.14.3)[13] to perform gene set analysis against the Gene Ontology annotation from org.Mm.eg.db R package (version 3.10.0). Data visualization was prepared using ggplot2 R package.

Reference-based annotation using SingleR. SingleR was used for reference-based annotation[14]. Data from Immgen was employed as reference to guide the annotation of $CD45^+$ immune cells sorted from the muscularis of WT mice[15]. For the annotation of the mono/Mφ subsets from WT and $CCR2^{-/-}$ mice, the manually curated annotations from the monocyte/Mφ subpopulations of Fig. 2 was used as reference.

Gene Set Enrichment Analysis. Gene Set Enrichment Analysis (GSEA) was performed using the GSEA software from the Broad Institute (version 4.0.2). Normalized counts of bulk RNA sequencing from $PLP1^+$ cells from the muscularis of $PLP1^{CreERT2}$ $Rpl22^{HA}$ mice at homeostasis and 3h after the induction of muscularis inflammation were used. Only genes that were significantly upregulated in the HA-tagged sample over the IgG control (adjusted p value cut off < 0.05 and log2(fold change) > 0.05) were used for the analysis. Extra data visualization was prepared using ggplot2 R package.

Supplementary figures:

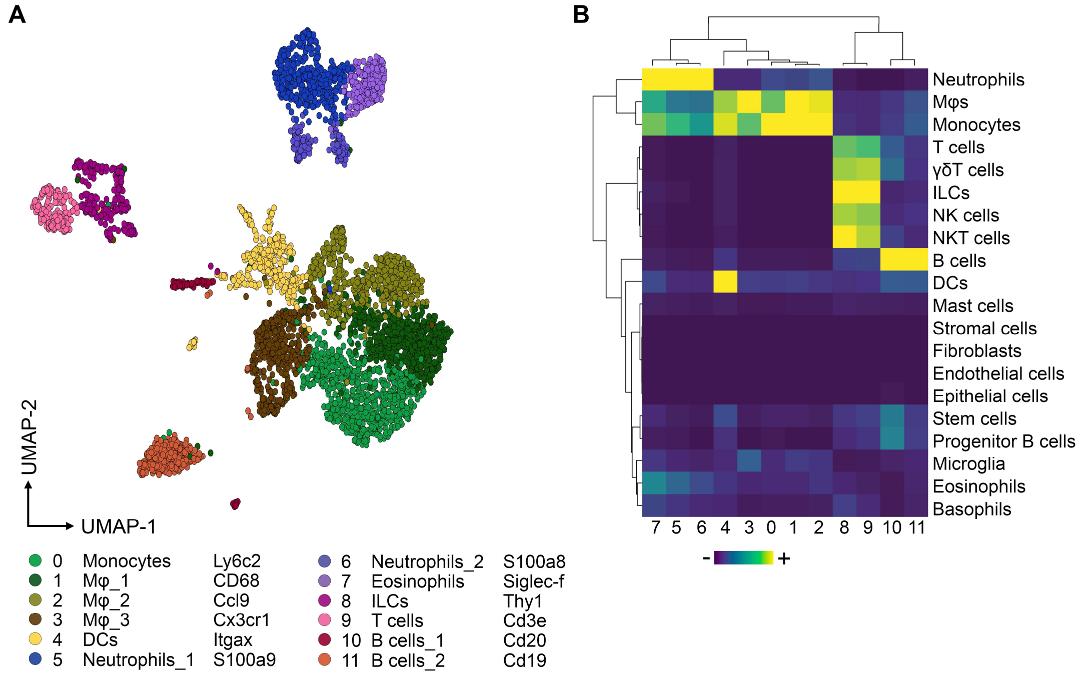

Supplementary Figure 1: SingleR annotation of CD45⁺ immune cells in the healthy and inflamed muscularis. A. UMAP of sorted CD45⁺ immune cells from the healthy muscularis, 24h and 72h after the induction of muscularis inflammation. Reproduction of Figure 1B. **B.** Automated SingleR annotation of clusters from Supplementary Fig. 1A using ImmGen as a reference database.

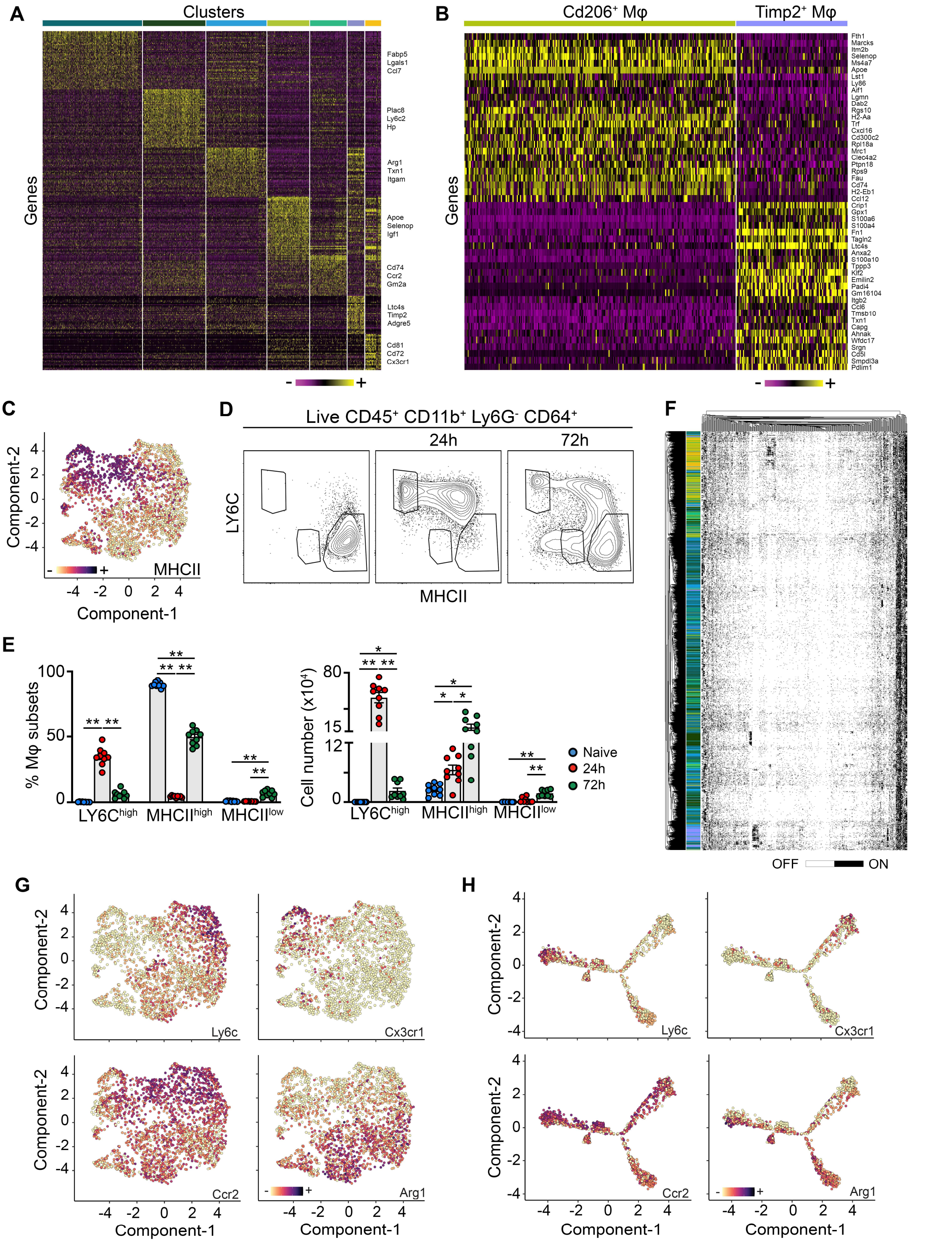

Supplementary Figure 2: Marker expression in myeloid sub-clustering. A. Heatmap of 50 most differentially expressed genes in each cluster from Fig. 2A. **B.** Heatmap of 25 most differentially expressed genes between Cd206$^+$ and Timp2$^+$ Mφs. **C.** UMAP depicting the expression of the MHCII gene signature. **D-E.** Immune cells isolated from the muscularis at homeostasis, 24h and 72h after muscularis inflammation were analyzed via flow cytometry. Representative contour plots showing Ly6C^{hi} monocytes, and Ly6C$^-$MHCII^{lo} Mφs and Ly6C$^-$MHCII^{hi} Mφs in the live CD45$^+$ CD11b$^+$ Ly6G$^-$ CD64$^+$ population (D). Percentage and absolute number of Ly6C^{hi} monocytes, Ly6C$^-$MHCII^{lo} Mφs and Ly6C$^-$MHCII^{hi} Mφs at different time points after muscularis inflammation. Data are shown as mean ± SEM. One-way ANOVA. *$p<0.05$; **$p<0.01$. (E). **F.** Hierarchically clustered heatmap showing 'binarized' regulon activity (ON/OFF indicated in Black/White) in each cell. Cells are annotated with the same colors as indicated in Figure 2A. **G.** Single cell gene expression of indicated markers projected onto a UMAP. **H.** Single cell gene expression of indicated markers projected onto the Monocle trajectory.

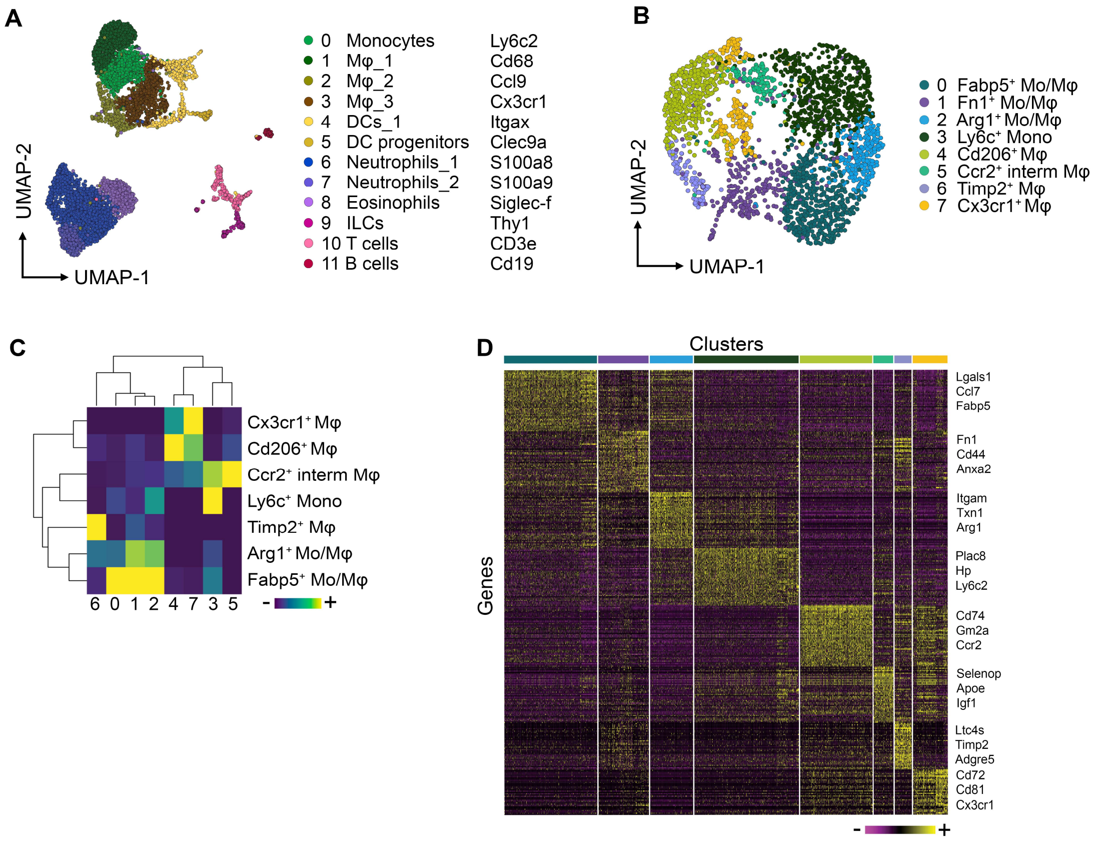

Supplementary Figure 3: Analysis of myeloid cells in CCR2$^{-/-}$ mice during muscularis inflammation. A-D. scRNAseq on sorted CD45^{+} immune cells from WT and CCR2$^{-/-}$ mice 24h and 72h after the induction of muscularis inflammation. **A.** UMAP of sorted CD45^{+} immune cells. **B.** UMAP of myeloid subclusters. **C.** Automated SingleR annotation of subclusters from Supplementary Fig. 3B based on the gene signature of subclusters from Fig. 2A. **D.** Heatmap of 50 most differentially expressed genes in each subcluster from Supplementary Fig. 3B.

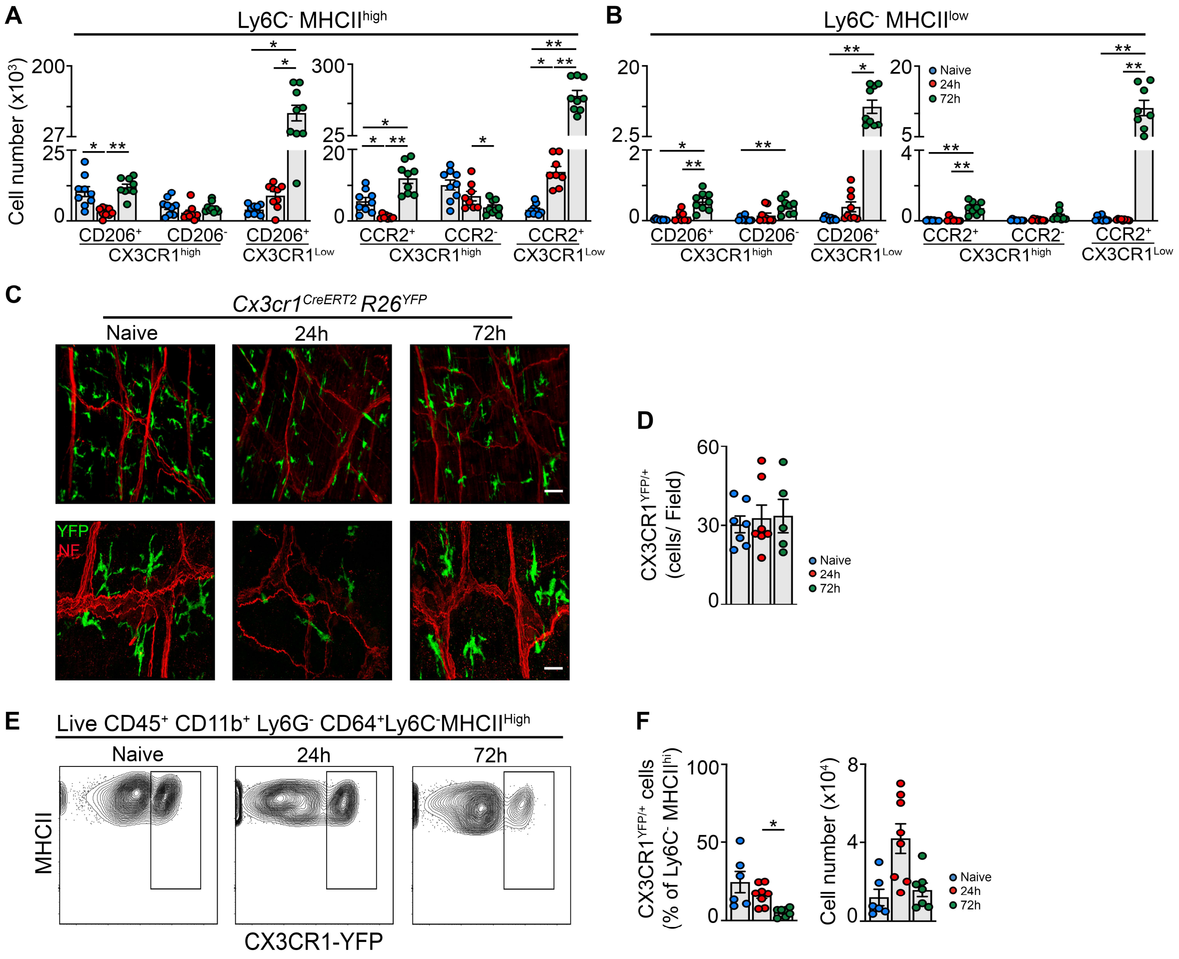

Supplementary Figure 4: Resident macrophages are not depleted upon muscularis inflammation. A-B. Analysis of absolute number of CD206$^+$ CX3CR1^{hi}, CD206$^-$ CX3CR1^{hi} and CD206$^+$ CX3CR1^{lo} cells in Ly6C$^-$ MHCII^{hi} Mφs (A) and Ly6C$^-$ MHCII^{lo} Mφs (B) and the absolute number of CCR2$^+$ CX3CR1^{hi}, CCR2$^-$ CX3CR1^{hi} and CCR2$^+$ CX3CR1^{lo} cells in the Ly6C$^-$ MHCII^{hi} Mφs (A) and Ly6C$^-$ MHCII^{lo} Mφs (B) from Fig. 4C,F. **C-F.** Analysis of the number of resident YFP$^+$ Mφs during muscularis inflammation (naïve, 24h and 72h) using tamoxifen-injected CX3CR1$^{CreERT2/+}$ Rosa26-LSL-YFP mice. **C.** Representative immunofluorescent images of muscularis whole mount preparations at different time points after muscularis inflammation, stained for YFP (green) and neurofilament (red). Scale bar 40 µm (top; 25X); 15 µm (bottom; 63X). **D.** Number of CX3CR1-YFP$^+$ cells per field. Each dot represents the average from an individual mouse and mean ± SEM are shown. Data are combined from 2 independent experiments. **E.** Contour plots showing CX3CR1-YFP expression in Ly6C$^-$ MHCII^{hi} Mφs at homeostasis and 24h and 72h after muscularis inflammation. **F.** Percentage of CX3CR1$^{YFP/+}$ cells in Ly6C$^-$ MHCII^{hi} Mφs (left) and absolute number of cells (right) at different time points after muscularis inflammation. A, B, D, F; one-way ANOVA. *p<0.05; **p<0.01, ns= not significant.

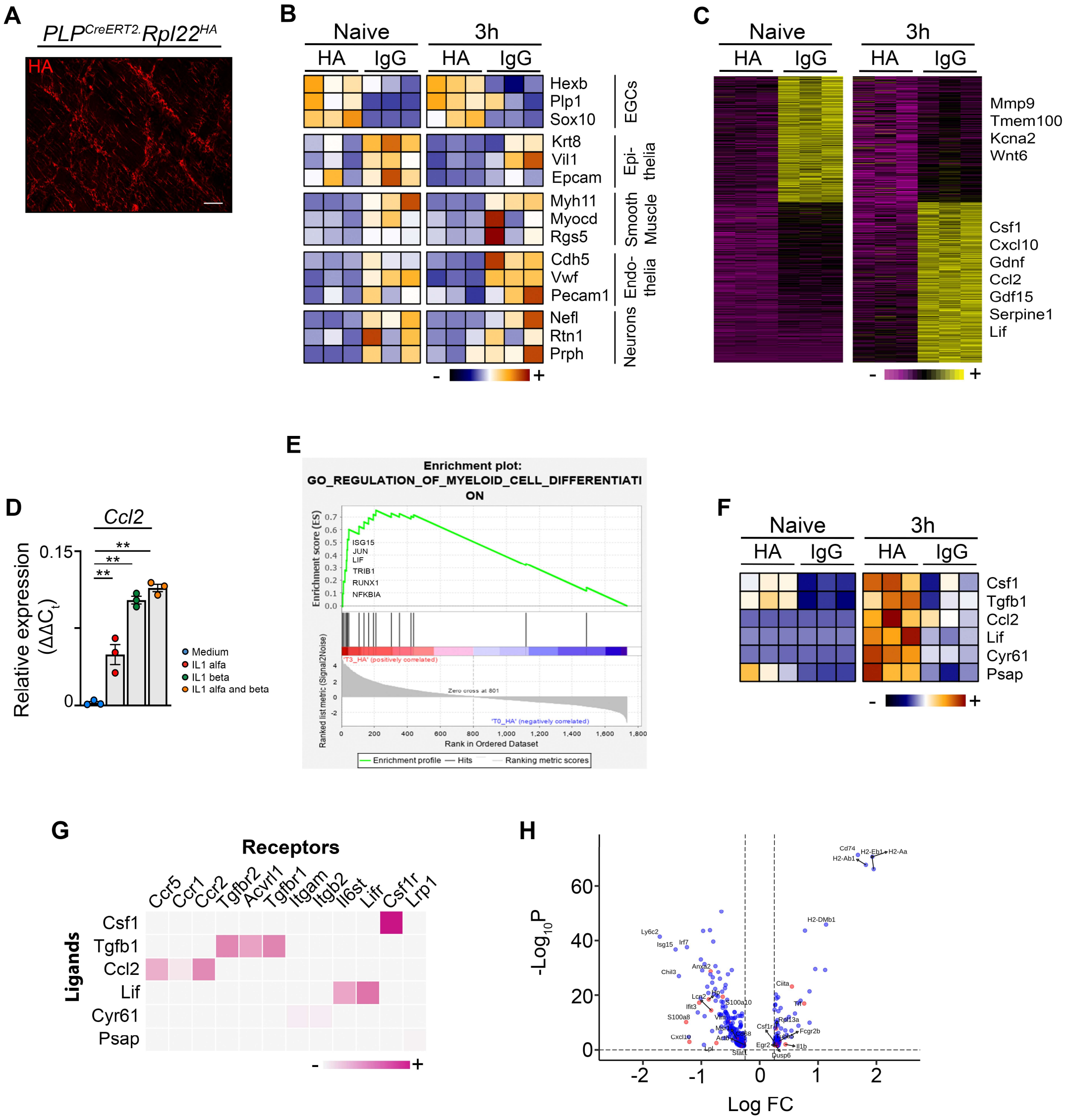

Supplementary Figure 5: Characterization of EGCs at homeostasis and during muscularis inflammation. A. Immunofluorescent image of muscularis whole mount preparation of a tamoxifen-injected PLP$^{CreERT2/+}$ Rpl22^{HA} mouse stained for HA tag (red). Scale bar 30 μm. **B.** Heat map of gene expression from IgG control or immunoprecipitated muscularis from naive mice or 3h after the induction of muscularis inflammation showing genes characteristics for different cell types. **C.** Heatmap of differentially expressed genes between HA immunoprecipitated samples from naïve PLP^{CreERT2} Rpl22^{HA} mice and 3h after intestinal manipulation compared with IgG control samples. **D.** Relative mRNA levels for Ccl2 normalized to the housekeeping gene rpl32 from neurosphere-derived EGCs stimulated with control, IL1-α and/or IL1-β for 18 hours. One-way ANOVA. **$p<0.01$. **E.** Visualization of GSEA analysis for regulation of myeloid cell differentiation. **F.** Heatmap of all positively correlated upstream ligands from NicheNet analysis from naïve PLP^{CreERT2} Rpl22^{HA} mice and 3h after intestinal manipulation compared with IgG control samples. **G.** Heatmap showing prior interaction potential between the ligands and corresponding receptors. **H.** Volcano plot of differentially expressed genes between Ccr2^{+} int Mφs and Ly6c^{+} monocytes highlighting target genes of top ligands from NicheNet in Ly6c^{+} monocytes and Ccr2^{+} int Mφs.

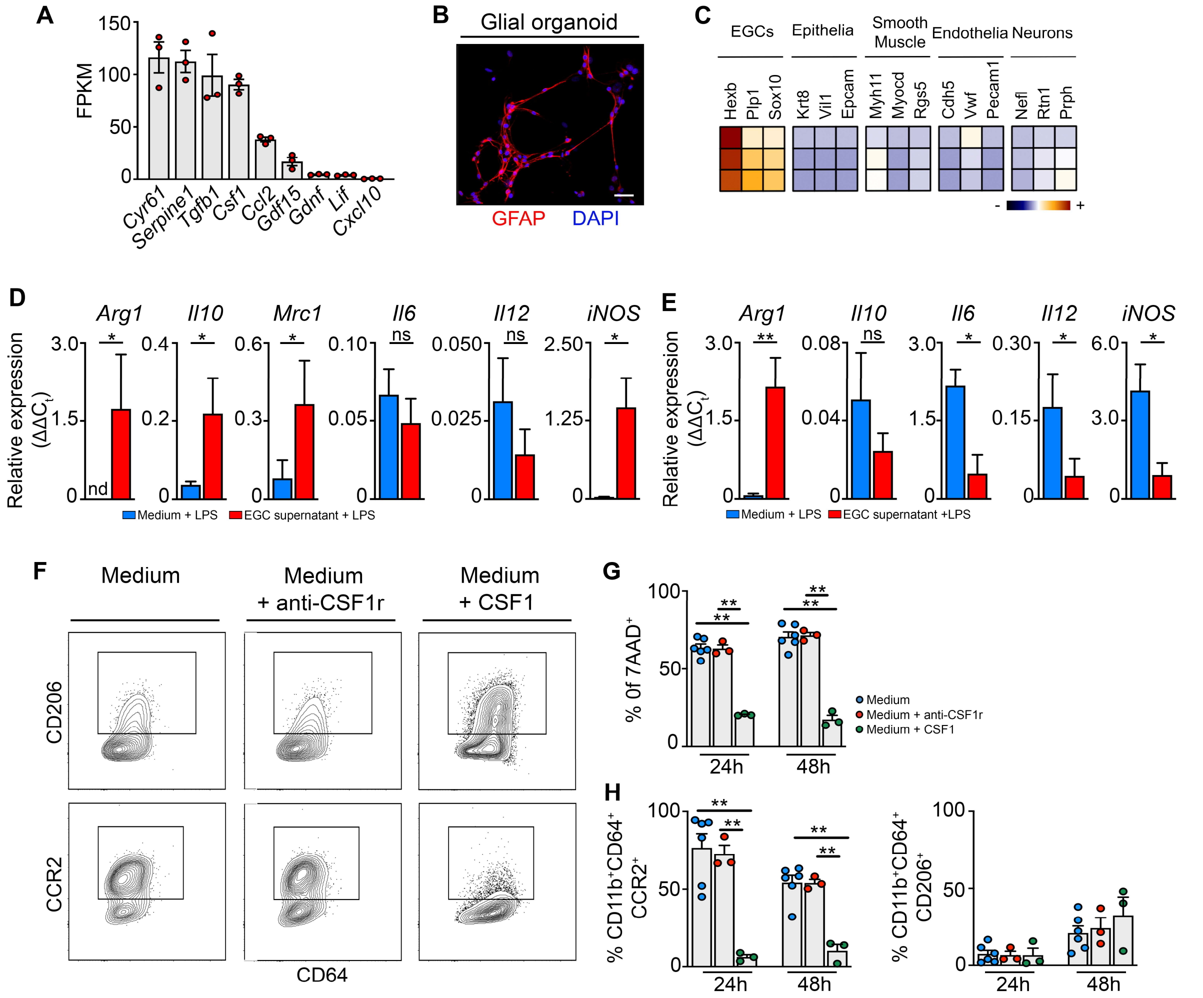

Supplementary Figure 6: The differentiation of monocytes into CD206^{+} Mφs is promoted by enteric glial cells *in vitro*, which is partly dependent upon CSF-1. A. FPKM values of bulk RNAseq performed on unstimulated neurosphere-derived EGCs. **B.** Neurosphere-derived EGCs stained with GFAP (red) and DAPI (blue). Scale bar 40 µm. **C.** Heatmap of genes specific for EGCs, epithelia, smooth muscle, endothelia and neurons in neurosphere-derived EGCs. **D.** Bone marrow-derived monocytes were cultured for 18h with/without EGC supernatant followed by LPS stimulation (100 ng/mL) for 6h. Relative mRNA levels for pro- and anti-inflammatory cytokines normalized to the housekeeping gene *rpl32* are shown. **E.** Ly6C^{hi} monocytes were sorted from the muscularis 24 post-injury and cultured 18h with/without EGC supernatant followed by LPS (100 ng/mL) for 6h. Relative mRNA levels for pro- and anti-inflammatory cytokines normalized to the housekeeping gene *rpl32* are shown. **F-H.** Bone marrow monocytes were cultured for 24-48h with medium alone, supplemented with anti-CSF1r (5 µg/mL) or CSF-1 (50 ng/mL). **F.** Contour plots of the expression of CD206 (top) and CCR2 (bottom) at 48h of culture. **G.** Quantification of the percentage of 7-AAD^{+} cells in bone marrow monocytes cultured for 24h and 48h. **H.** Percentage of CCR2^{+} and CD206^{+} cells in the live CD45^{+} CD11b^{+} Ly6G^{-} CD64^{+} population.

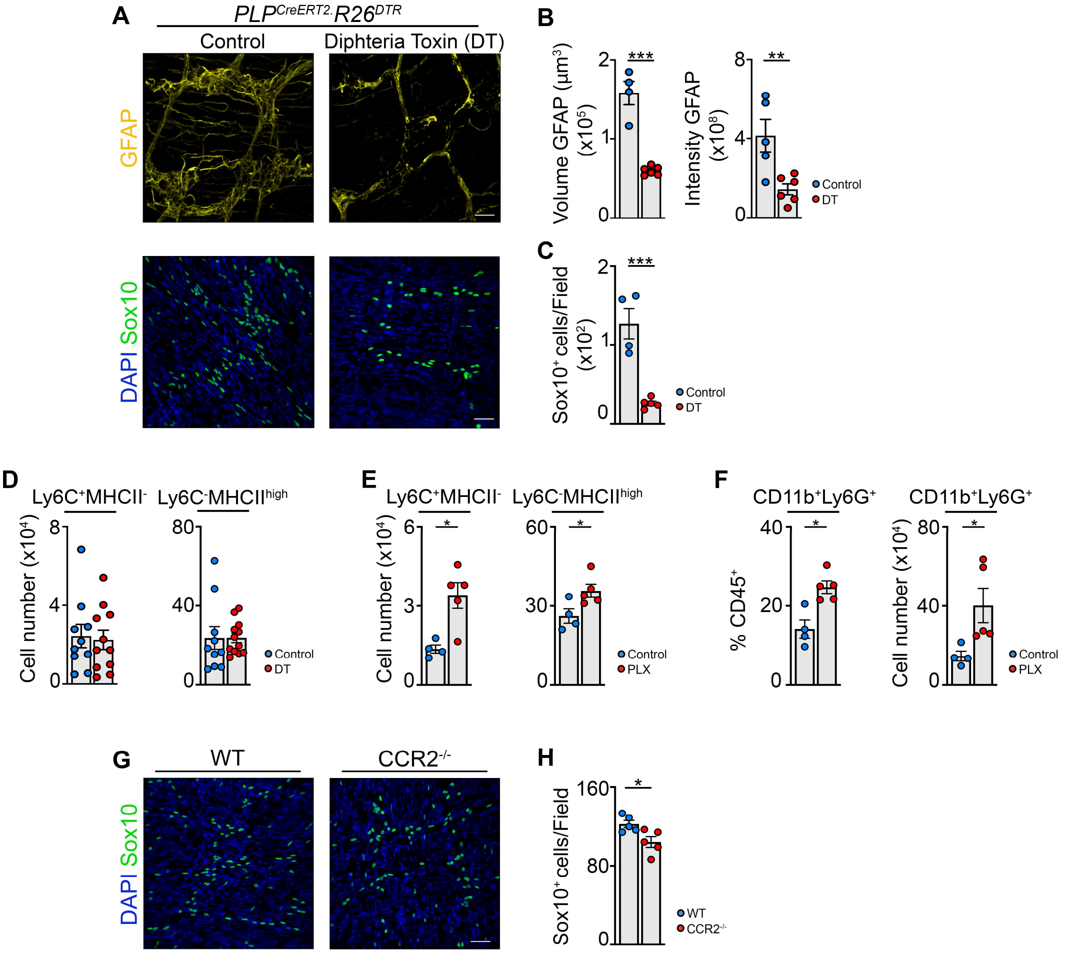

Supplementary Figure 7: Enteric glial cells play a crucial role in the differentiation of monocytes in $CD206^+$ Mφs during muscularis inflammation, which is partly dependent upon CSF-1. A. Immunofluorescent images of muscularis whole mount preparations of control and DT treated $PLP^{CreERT2/+}$ iDTR mice 72h after muscularis inflammation and stained for GFAP (yellow; top) or SOX10 (green) and DAPI (blue; bottom). Scale bar 30 µm. **B.** Quantification of the volume (left) and intensity (right**)** of GFAP (N=4-5 pictures/mouse). **C.** Quantification of number of $Sox10^+$ EGCs (N=4-5 pictures/mouse). **D.** Analysis of the absolute number of $Ly6C^+$ $MHCII^-$ monocytes and $Ly6C^-$ $MHCII^{hi}$ Mφs in control and DT treated $PLP^{CreERT2/+}$ iDTR mice 72h after muscularis inflammation. **E.** Analysis of the absolute number of $Ly6C^+$ $MHCII^-$ monocytes and $Ly6C^-$ $MHCII^{hi}$ Mφs in control and PLX-3397 treated mice 72h after muscularis inflammation. **F.** Percentage (left) and cell number (right) of infiltrating $CD11b^+$ $Ly6G^+$ neutrophils in the muscularis of control and PLX treated mice 72h after muscularis inflammation. **G.** Immunofluorescent image of muscularis whole mounts of WT and $CCR2^{-/-}$ mice 72h after muscularis inflammation and stained for SOX10 (green) and DAPI (blue). Scale bar 30 µm. **H.** Quantification of the number of $SOX10^+$ cells per field (N=4-5 pictures/mouse). B-F, H. t-test. *p<0.05; **p<0.01.

References

1. van Bree SH, Nemethova A, van Bovenkamp FS, et al. Novel method for studying postoperative ileus in mice. Int J Physiol Pathophysiol Pharmacol 2012;4:219-27.
2. Lennon VA, Sas DF, Busk MF, et al. Enteric neuronal autoantibodies in pseudoobstruction with small-cell lung carcinoma. Gastroenterology 1991;100:137-42.
3. De Schepper S, Verheijden S, Aguilera-Lizarraga J, et al. Self-Maintaining Gut Macrophages Are Essential for Intestinal Homeostasis. Cell 2018;175:400-415 e13.
4. Soret R, Coquenlorge S, Cossais F, et al. Characterization of human, mouse, and rat cultures of enteric glial cells and their effect on intestinal epithelial cells. Neurogastroenterol Motil 2013;25:e755-64.
5. Mich JK, Signer RA, Nakada D, et al. Prospective identification of functionally distinct stem cells and neurosphere-initiating cells in adult mouse forebrain. Elife 2014;3:e02669.
6. Livak KJ, Schmittgen TD. Analysis of relative gene expression data using real-time quantitative PCR and the 2(-Delta Delta C(T)) Method. Methods 2001;25:402-8.
7. Haimon Z, Volaski A, Orthgiess J, et al. Re-evaluating microglia expression profiles using RiboTag and cell isolation strategies. Nat Immunol 2018;19:636-644.
8. Kim JS, Kolesnikov M, Peled-Hajaj S, et al. A Binary Cre Transgenic Approach Dissects Microglia and CNS Border-Associated Macrophages. Immunity 2021;54:176-190 e7.
9. Jaitin DA, Kenigsberg E, Keren-Shaul H, et al. Massively parallel single-cell RNA-seq for marker-free decomposition of tissues into cell types. Science 2014;343:776-9.
10. Kohen R, Barlev J, Hornung G, et al. UTAP: User-friendly Transcriptome Analysis Pipeline. BMC Bioinformatics 2019;20:154.
11. Love MI, Huber W, Anders S. Moderated estimation of fold change and dispersion for RNA-seq data with DESeq2. Genome Biol 2014;15:550.
12. Aibar S, Gonzalez-Blas CB, Moerman T, et al. SCENIC: single-cell regulatory network inference and clustering. Nat Methods 2017;14:1083-1086.
13. Yu G, Wang LG, Han Y, et al. clusterProfiler: an R package for comparing biological themes among gene clusters. OMICS 2012;16:284-7.
14. Aran D, Looney AP, Liu L, et al. Reference-based analysis of lung single-cell sequencing reveals a transitional profibrotic macrophage. Nat Immunol 2019;20:163-172.
15. Heng TS, Painter MW, Immunological Genome Project C. The Immunological Genome Project: networks of gene expression in immune cells. Nat Immunol 2008;9:1091-4.
